# Supplementary material for: Nucleoporin 107, 62 and 153 mediate Kcnq1ot1 imprinted domain regulation in extraembryonic endoderm stem cells
Source: Nat Commun. 2018 Jul 18;9:2795. doi: 10.1038/s41467-018-05208-2 (PMC6052020; doi:10.1038/s41467-018-05208-2)
Supplement: Supplementary file 1 — Supplementary Information [file 41467_2018_5208_MOESM1_ESM.pdf]

Supplemental Information

Nucleoporin 107, 62 and 153 mediate *Kcnq1ot1* imprinted domain regulation in extraembryonic endoderm stem cells

Sachani et. al

Inventory

Supplementary Figures and Legends 1-38
Supplementary Tables 1-5
Supplemental Methods
Supplemental References

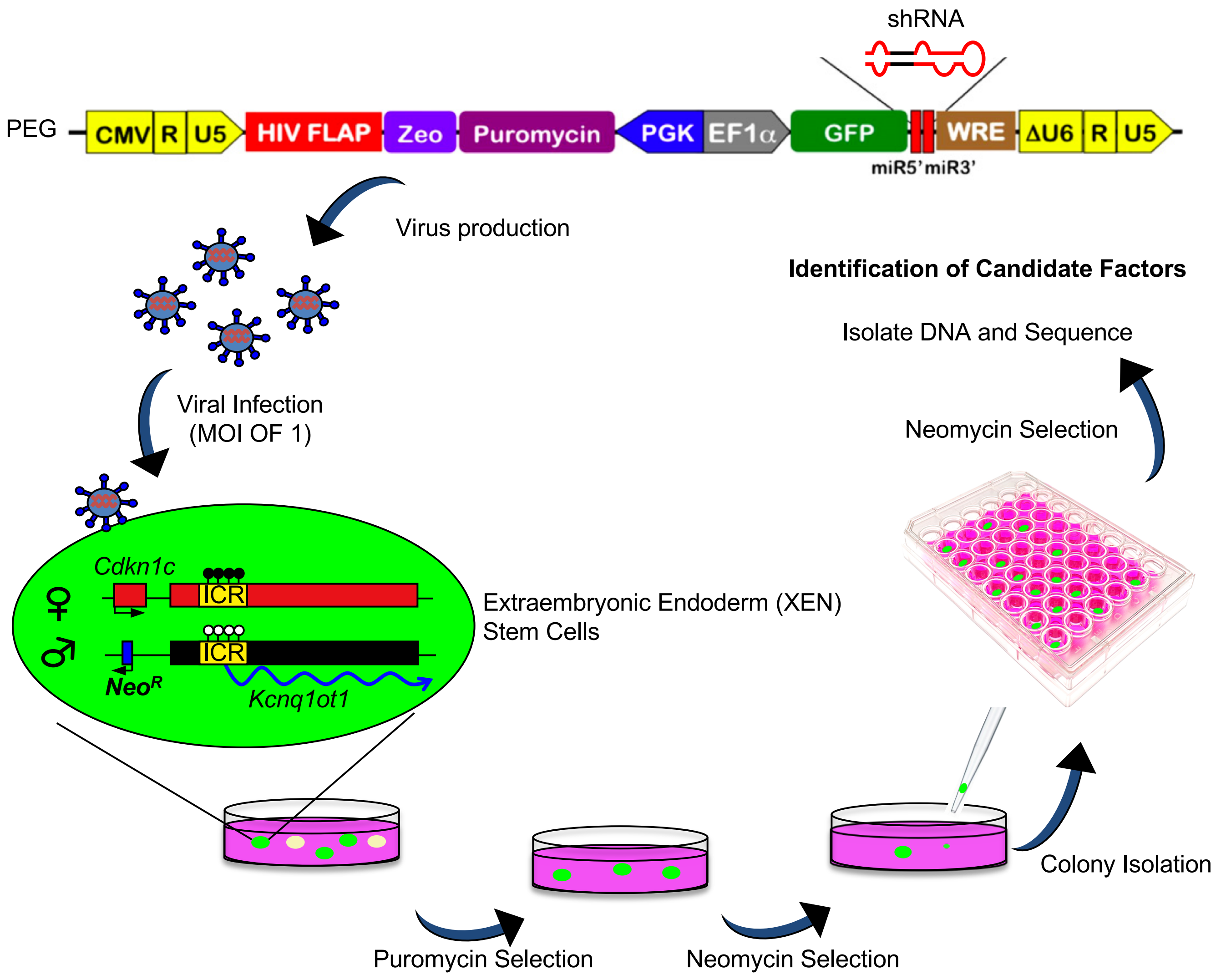

Supplementary Figure 1: Positive-selection, loss-of-function RNA interference screen for epigenetic factors regulating the *Kcnq1ot1* imprinted domain.
Low multiplicity lentivirus infection was carried out in paternal *Cdkn1c*$^{+\Delta neoR}$ XEN cells using a second-generation RNAi library (~750 shRNA^{miR}s) targeting 250 known or putative epigenetic factors, which was engineered within the bidirectional PGK-EF1α-GFP (PEG) lentiviral delivery system (Golding et. al., 2010). The epigenetic library was divided into four pools, allowing for independent rounds of screening. Puromycin selection allowed cells with an integrated shRNA to survive. RNA depletion of epigenetic factors which caused reactivation of the paternally-silenced neoR cassette permitted selection of cells when neomycin was added to the culture media. Following the first round of neomycin selection, colonies were picked and expanded in a 24-well dish. Following a second-round of neomycin selection, candidate factors were identified by DNA sequencing.

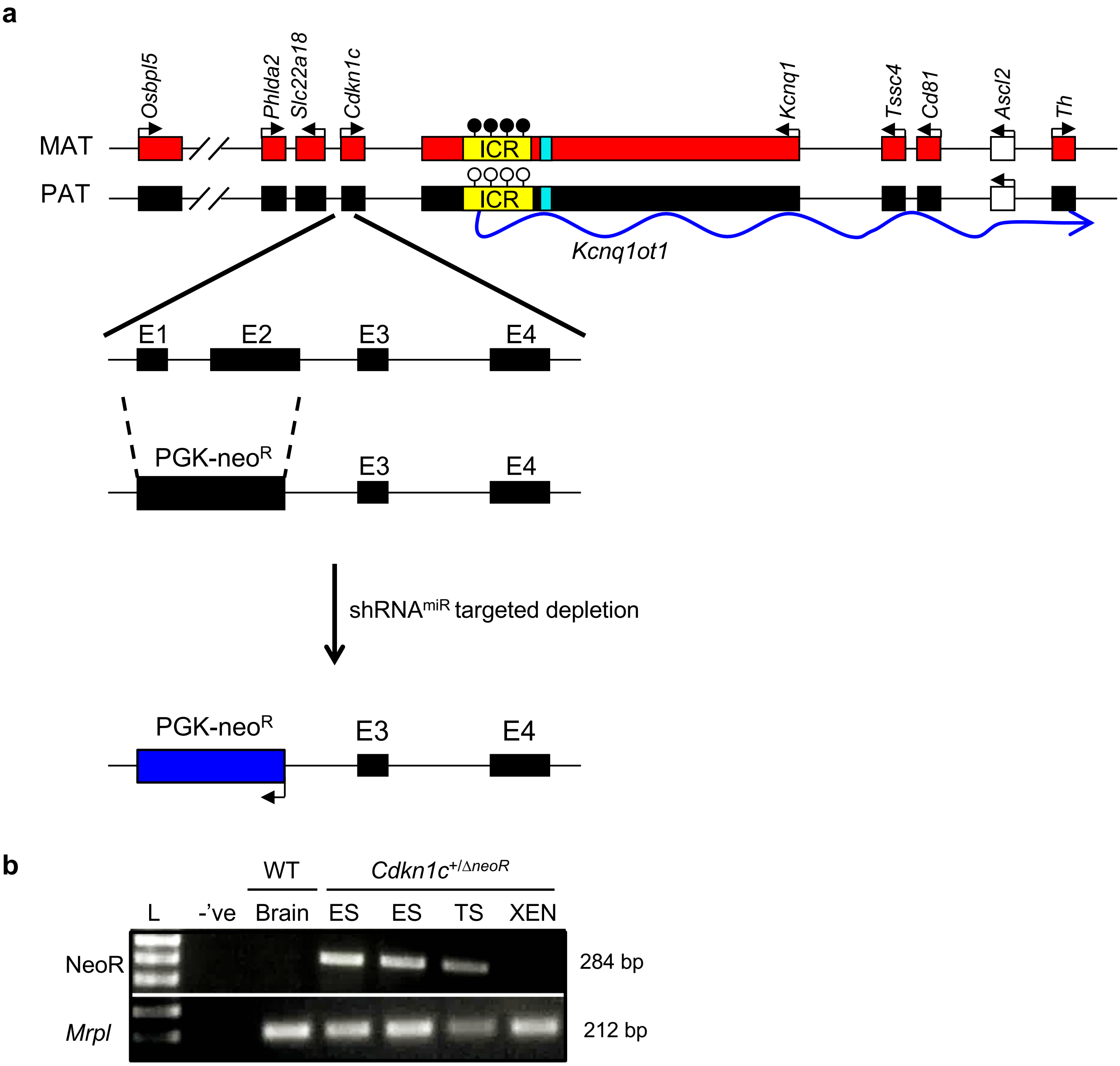
a
Osbpl5
Phlda2
Slc22a18
Cdkn1c
Kcnq1
Tssc4
Cd81
Ascl2
Th
MAT
PAT
ICR
ICR
Kcnq1ot1
E1
E2
E3
E4
PGK-neoR
E3
E4
shRNAmiR targeted depletion
PGK-neoR
E3
E4
b
WT
Cdkn1c+/ΔneoR
L
-'ve
Brain
ES
ES
TS
XEN
NeoR
Mrpl
284 bp
212 bp

Supplementary Figure 2: NeoR gene recombined at *Cdkn1c* was silent on the paternal allele at the *Kcnq1ot1* domain in XEN cells.

(**a**) Strategy for reactivation of the paternally-inherited neomycin resistance (NeoR) gene upon depletion of targeted epigenetic factors. In a previously generated gene-targeted mouse mutant, the first two exons of the *Cdkn1c* gene were deleted and replaced with the PGK-driven neomycin resistance cassette ($Cdkn1c^{\Delta neoR}$) (Zhang et al., 1997). Like *Cdkn1c*, paternal inheritance of the $Cdkn1c^{\Delta neoR}$ allele results in NeoR gene silencing in midgestation embryos and placenta (Caspary et., 1998). Thus, targeted depletion of epigenetic factors by shRNAs, which results in reactivation of $Cdkn1c^{\Delta neoR}$, will allow for positive selection of colonies. (**b**) The paternally-inherited NeoR gene was silenced in XEN cells. NeoR expression was detected by RT-PCR in CAST7XB6 $Cdkn1c^{+/\Delta neoR}$ ES and TS cells but not in the negative control wildtype (WT) brain. By comparison, NeoR gene was repressed in CAST7X B6 $Cdkn1c^{+/\Delta neoR}$ XEN cells. NeoR expression was normalized to mitochondrial ribosomal protein L1 (*Mrpl*). Note that maternal transmission of $Cdkn1c^{\Delta neoR}$ in XEN, ES and TS cells was not examined to assess whether it remained active when maternally inherited, similar to midgestation tissues (Caspary et al., 1998). L, ladder; -'ve, PCR negative control; ES, embryonic stem cells; TS, trophectoderm stem cells; XEN, extraembryonic endoderm stem cells. Full gel images are shown in Supplementary Figure 31.

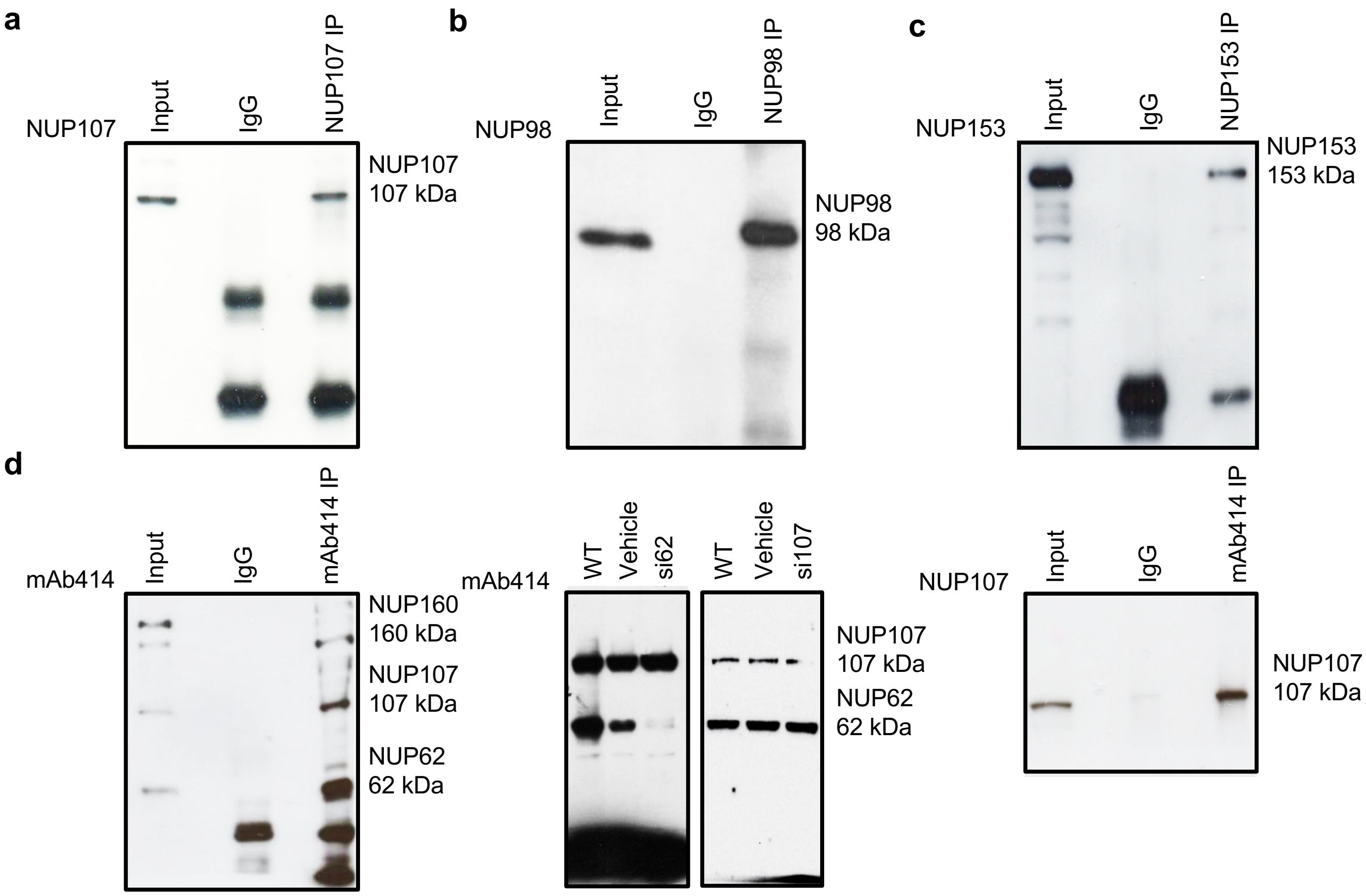

Supplementary Figure 3: Antibody validation in XEN cells.
(**a**) NUP107 IP was performed followed by Western blot analysis using the same NUP107 antibody. (**b**) NUP98 IP was performed followed by Western blot analysis using the same NUP98 antibody. (**c**) NUP153 IP was performed followed by Western blot analysis using the same NUP153 antibody. (**d**) mAb414 IP was performed followed by Western blot analysis using the same mAb414 antibody. The most prominent nucleoporins detected with the mAb414 antibody in XEN cells were NUP62, NUP107 and NUP160, as identified by size. NUP62 and NUP107 identity was additionally verified using *Nup62*-depleted and *Nup107*-depleted XEN cells using the mAb414 antibody. NUP107 band identity was also verified by mAb414 IP followed by Western blot analysis using the NUP107 antibody. Full Western blot images shown.

Sachani Supplementary Figure 4

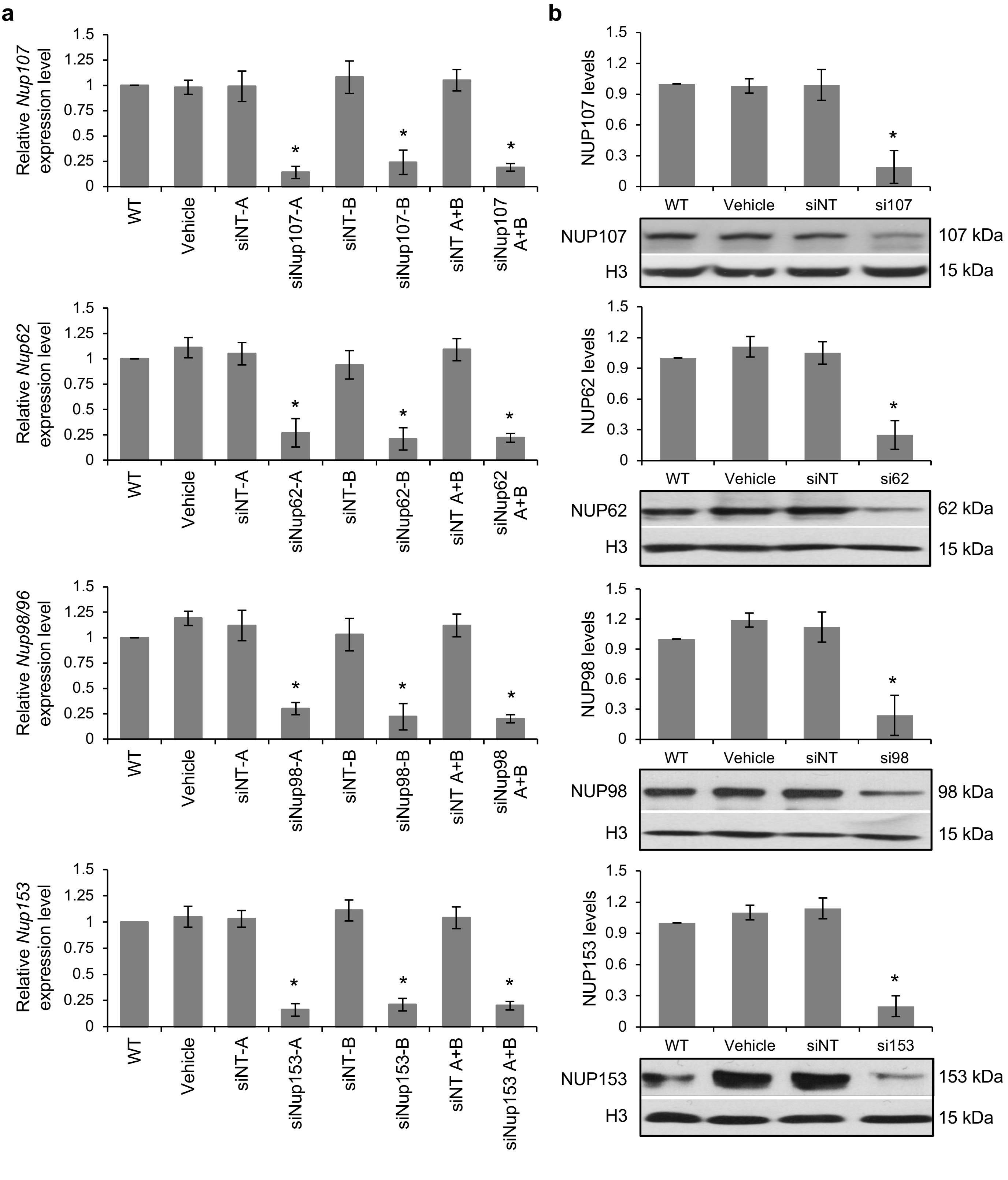

Supplementary Figure 4: Nucleoporin depletion levels in XEN cells.

(**a**) Nucleoporin RNA depletion levels. Quantitative real-time PCR analysis for *Nup107, Nup62, Nup98/96* and *Nup153* relative to *Gapdh* expression 48 hours after transfection. Transfections were performed using the two different sets of siRNAs, singly and together (A and B; n=3 biological samples with 3 technical replicates per sample). The *Nup98* gene is a bicistronic gene that encodes for two separate nucleoporins, NUP98 and NUP96, from one mRNA. Since the siRNAs targets the mRNA that will produce both proteins, the siRNAs have been designated si98/96. All subsequent transfections employed both sets (A and B) of siRNAs. (**b**) Nucleoporin protein depletion levels. Western blot analysis using NUP107, mAb414, NUP98 and NUP153 antibodies was performed 48 hours after transfection. The NUP98 antibody specifically recognized NUP98; no commercial antibody was available for NUP96. Histone 3 (H3) was used as loading control. Transfections were performed using the two different sets of siRNAs (n=6 biological samples with 3 technical replicates per sample). mRNA abundance and protein levels were depleted to 0.14/0.24 and 0.19 times for *Nup107*, to 0.27/0.21 and 0.25 times for *Nup62*, to 0.30/0.22 and 0.25 times for *Nup98/96,* and to 0.16/0.21 and 0.20 times for *Nup153,* respectively. Error bars indicate s.e.m.; *, significance $p < 0.05$ compared to the WT control; Veh, vehicle; siNT, non-targeting siRNA; si107, *Nup107* siRNA; si62, *Nup62* siRNA; si98/96, *Nup98/96* siRNA; si153, *Nup153* siRNA. Full Western blot images are shown in Figure 32.

Sachani Supplementary Figure 5

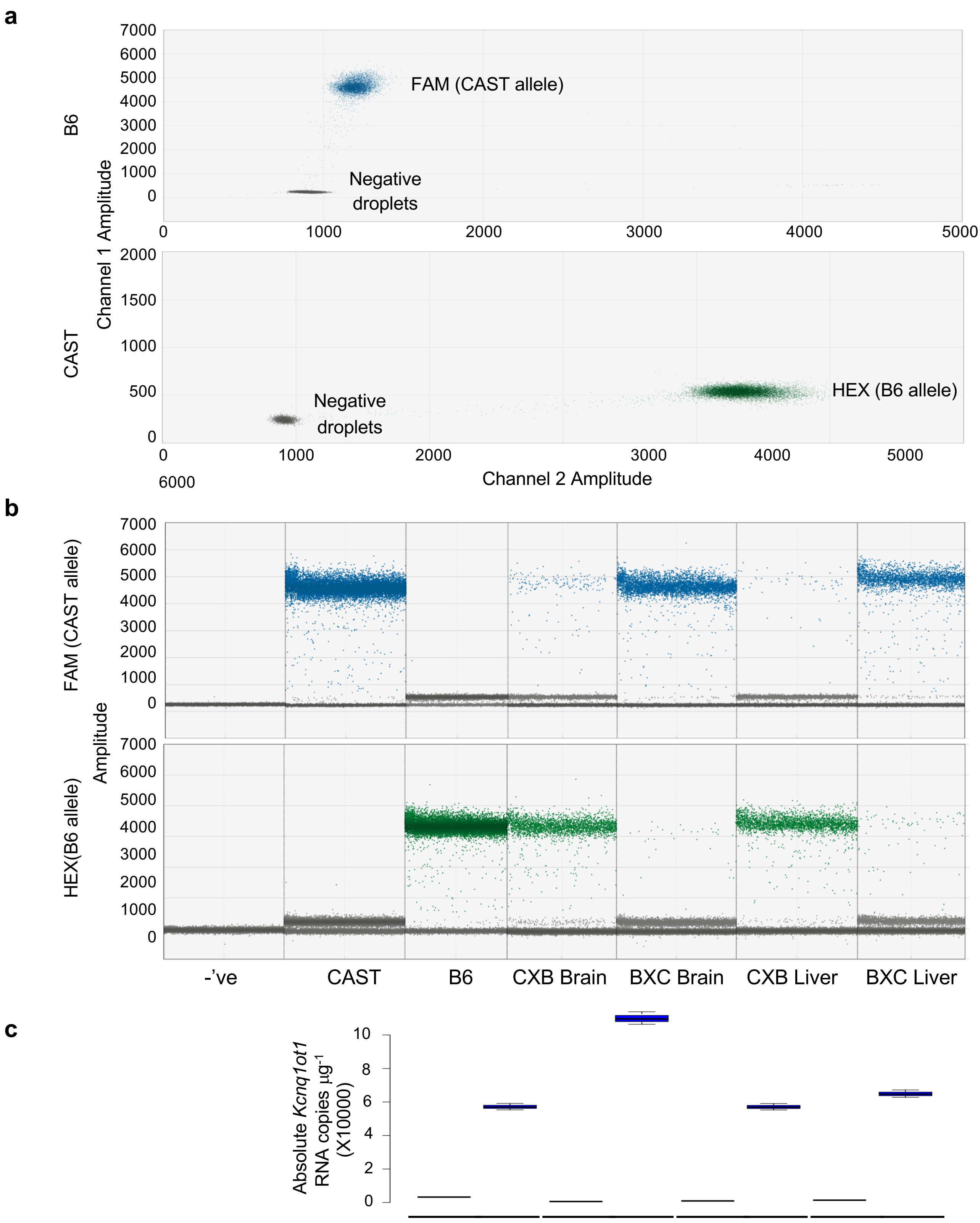

	CXB Brain	BXC Brain	CXB Liver	BXC Liver
Maternal	3281	612	965	1421
Paternal	57156	109921	57092	64880

Supplementary Figure 5: Droplet digital PCR analysis of *Kcnq1ot1* long non-coding RNA

(a) Two-dimensional droplet digital PCR assay for WT *Kcnq1ot1* shows expression of the gene primarily from the CAST allele detected by the FAM (blue) probe and the B6 allele detected by the HEX probe (green). **(b)** One-dimensional representation of the FAM and HEX droplets for *Kcnq1ot1* for CAST and B6 controls, and reciprocal F_1 brain and liver (female listed first in crosses). **(c)** Absolute quantification of B6 and CAST *Kcnq1ot1* RNA copies in 1 μg of RNA from samples in (b) using droplet digital PCR (n=3 technical replicates). Center lines, medians; box limits, 25th and 75th percentiles as determined by R software; whiskers, 1.5 times the interquartile range from 25th and 75th percentiles.

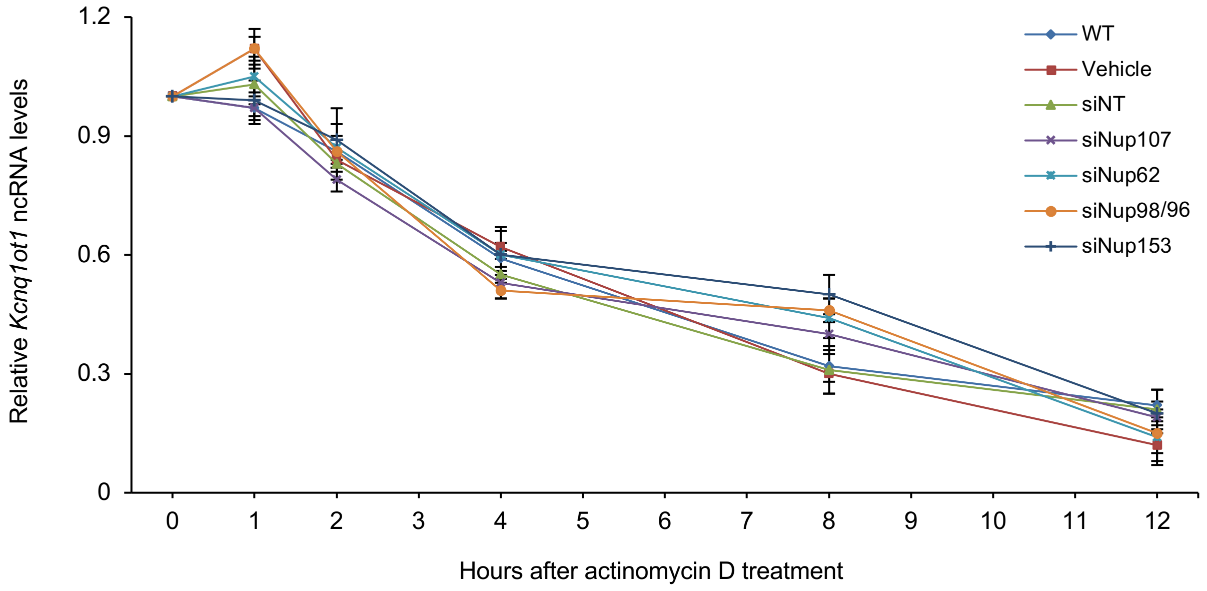

Supplementary Figure 6: *Kcnq1ot1* ncRNA stability was not altered upon nucleoporin depletion in XEN cells.
Control and *Nup*-depleted XEN cells were treated with actinomycin D for 1 hour, after which cells were collected up to 12 hours after released from treatment. *Kcnq1ot1* expression levels were normalized to 0 hours. No significant changes in *Kcnq1ot1* ncRNA levels were seen at different time intervals after treatment between samples compared to the WT control, indicating that there was no difference in *Kcnq1ot1* ncRNA half-life (5-6 hours) in control and *Nup*-depleted XEN cells (n=3 biological samples with 3 technical replicates per sample). Error bars, s.e.m.; *, significance $p < 0.05$ compared to the WT control; WT, wildtype; Veh, vehicle; siNT, non-targeting siRNA; si107, *Nup107* siRNA; si62, *Nup62* siRNA; si98/96, *Nup98/96* siRNA; si153, *Nup153* siRNA.

Sachani Supplementary Figure 7

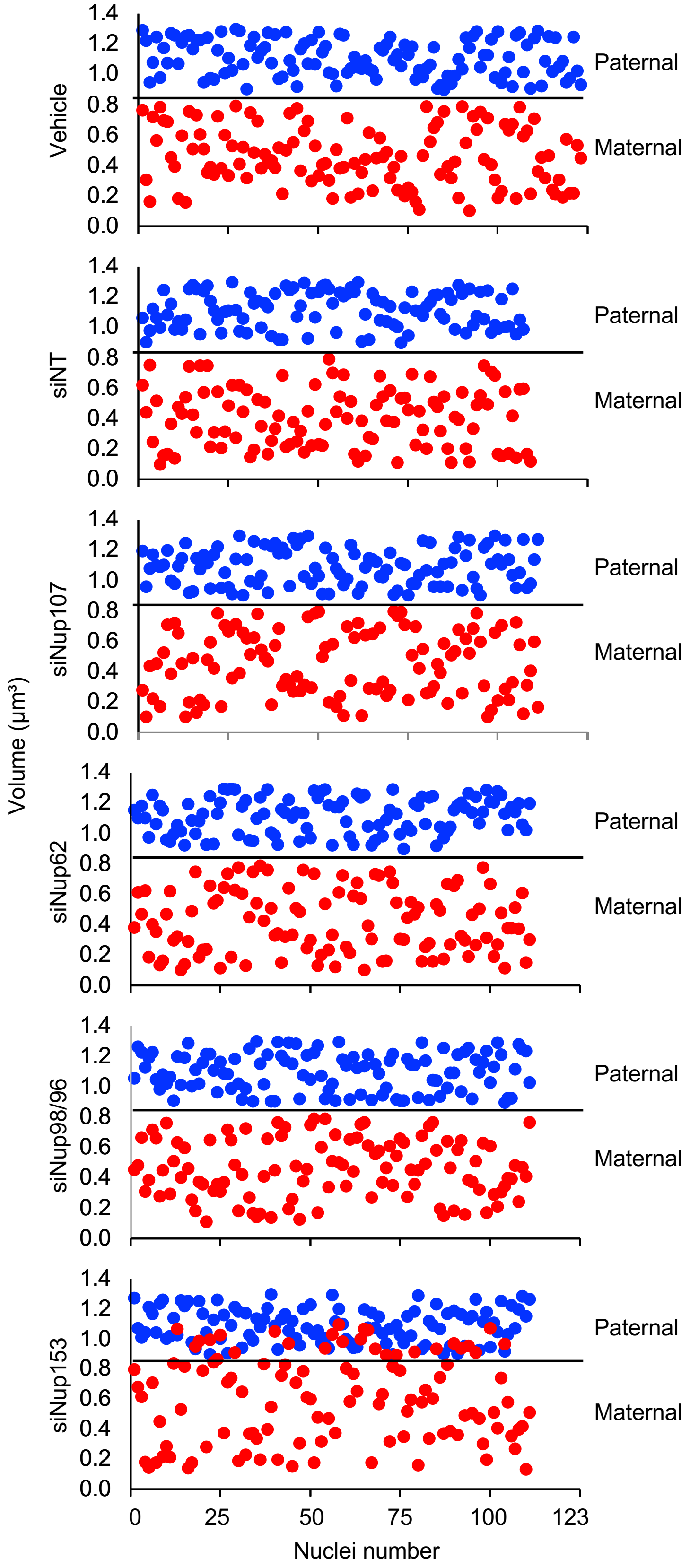

Supplementary Figure 7: Paternal and maternal *Kcnq1ot1* domain have distinct volumes in XEN cells.

Paternal (blue, identified by *Kcnq1ot1* ncRNA expression) and maternal (red) *Kcnq1ot1* domain volume were plotted on the Y-axis with the number of G1-synchronized control and *Nup*-depleted XEN cells plotted on the X-axis. Paternal *Kcnq1ot1* DNA domain volume had a range of 0.9 to 1.3 μm^3 while maternal *Kcnq1ot1* DNA domain volume ranged from 0.1 to 0.8 μm^3, except for the siNup153 treatment group, where the maternal *Kcnq1ot1* DNA domain volume ranged from 0.14 to 1.1 μm^3 (black bar, 0.85 μm^3). For those cells with a maternal *Kcnq1ot1* DNA domain volume greater than 0.9 μm^3, the majority (92%) possessed a equal or larger paternal *Kcnq1ot1* DNA domain volume; n=109-142. Nuclei with no DNA FISH signal were excluded (Vehicle, 5%; siNT, 7%; si107, 7%; si62, 5%; si98/96, 6%; si153, 5% of cells).

Sachani Supplementary Figure 8

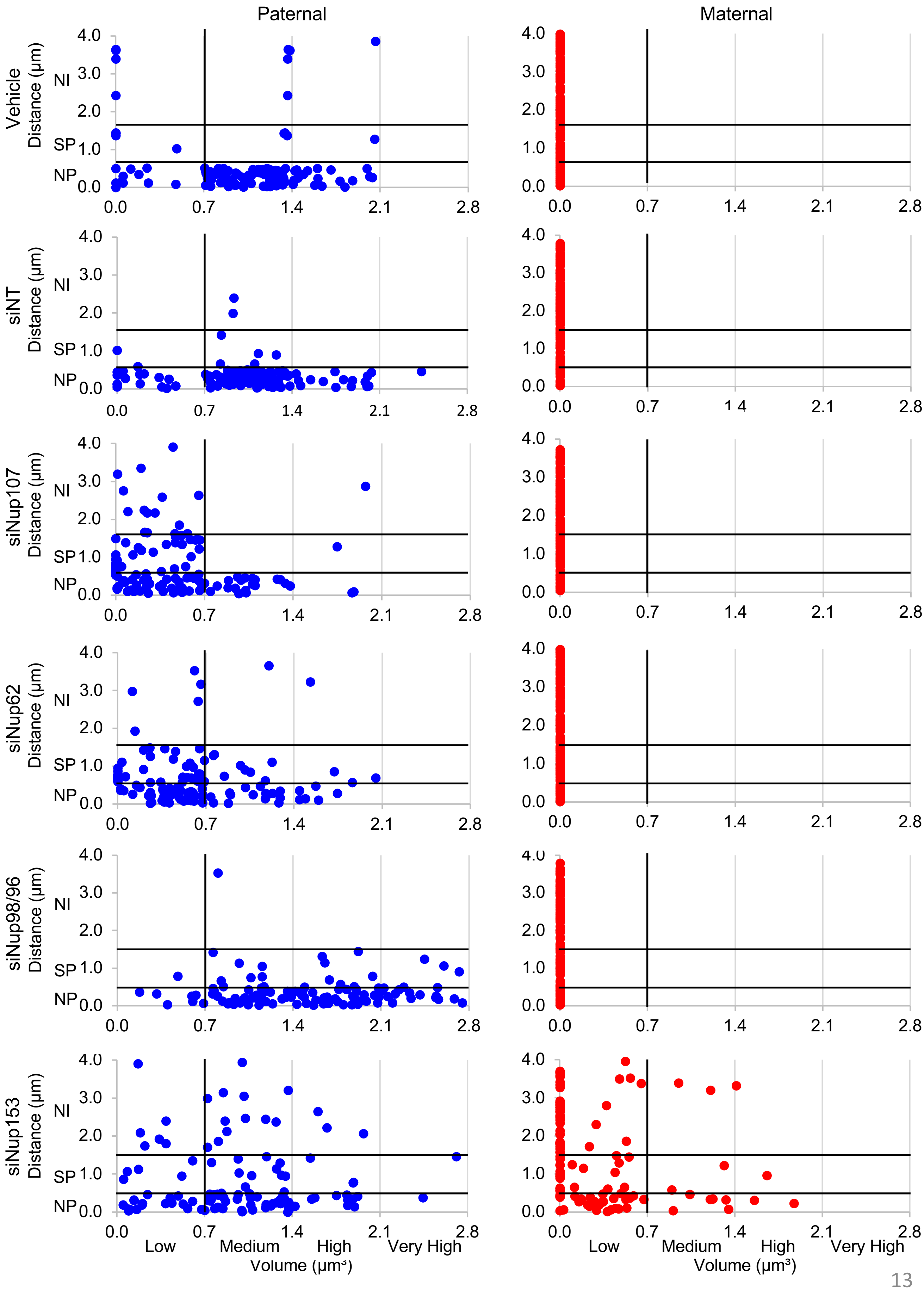

Supplementary Figure 8: *Kcnq1ot1* ncRNA volume to distance correlation in control and nucleoporin-depleted XEN cells.

Kcnq1ot1 ncRNA volume and domain distance from nuclear periphery were plotted on X- and Y-axes, respectively, for G1-synchronized control and *Nup*-depleted XEN cells. Upon *Nup107*, *Nup62* and *Nup153* depletion, cells with low paternal *Kcnq1ot1* ncRNA volume had a shift in paternal *Kcnq1ot1* domain positioning toward the sub-nuclear peripheral and nuclear interior. The maternal *Kcnq1ot1* domain was randomly positioned within the nucleus (expected NP 15%, SP 30%, NI 60%; observed NP 9-17%, 21-28; NI, 57-66), except for *Nup153*-depleted cells. Upon *Nup153* depletion, those cells with maternal *Kcnq1ot1* ncRNA expression (primarily low) had a shift in maternal *Kcnq1ot1* domain positioning toward the nuclear periphery (NP 60%, SP 21%, NI, 19%). NP, nuclear periphery; SP, sub-nuclear periphery; NI, nuclear interior; Veh, vehicle; siNT, non-targeting siRNA; si107, *Nup107* siRNA; si62, *Nup62* siRNA; si98/96, *Nup98/96* siRNA; si153, *Nup153* siRNA; n=109-142. Nuclei with no RNA and DNA FISH signal were excluded (Vehicle, 9%; siNT, 11%; si107, 9%; si62, 11%; si98/96, 12%; si153, 8% of cells).

Sachani Supplementary Figure 9

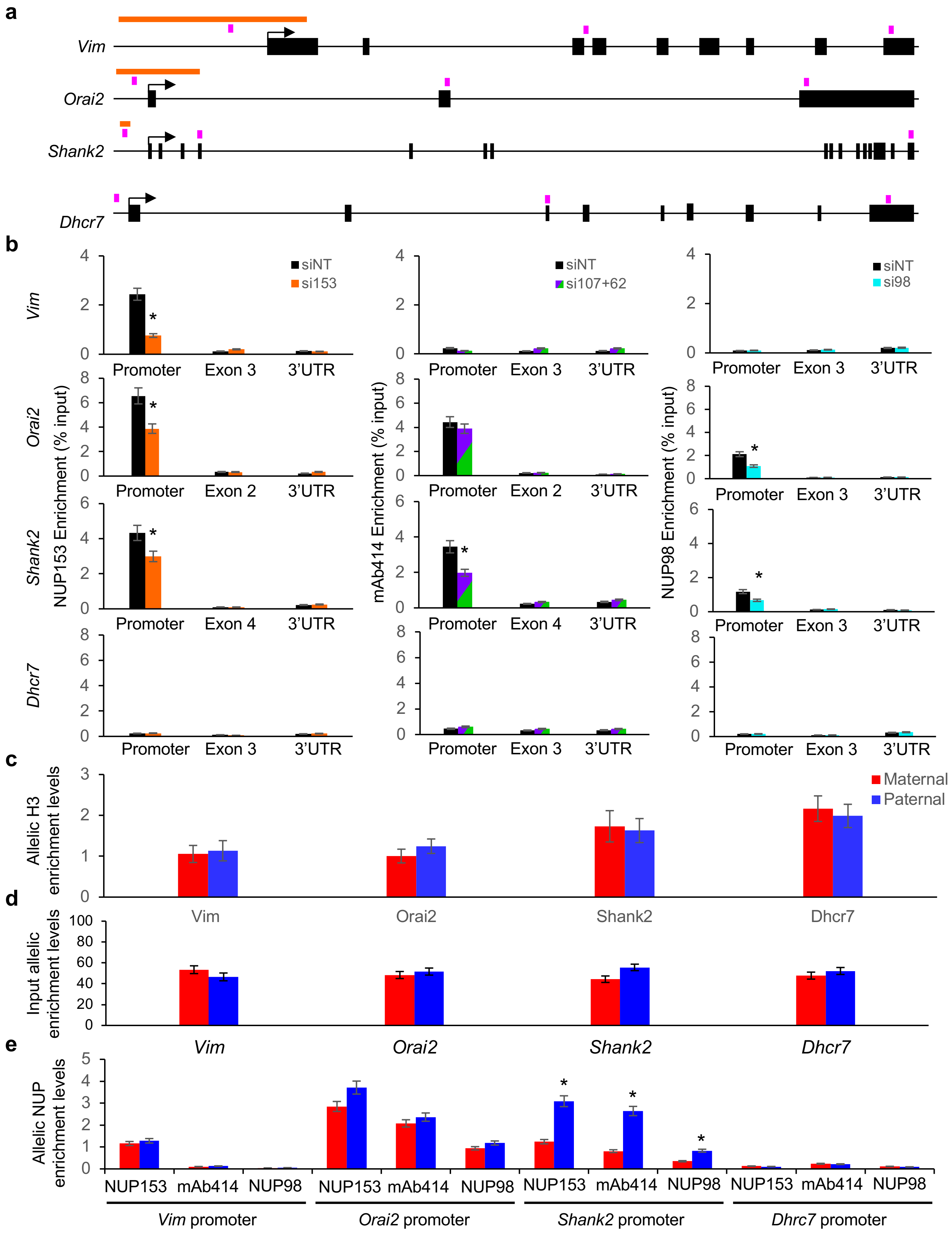

Supplementary Figure 9: mAb414 and NUP153 enrichment at the *Kcnq1ot1* ICR and enhancer element in XEN cells.

(**a**) NUP153 enrichment regions were mapped from ES cell DamID-seq data (Jacinto et. al, 2015) at the *Vim*, *Orai2* and *Shank2* promoters (orange bar). These genes were not upregulated upon *Nup153* depletion in ES cells and/or in ES cells differentiated into NeuP cells (Jacinto et. al, 2015). Positive sites within the enriched region as well as negative control sites within *Vim*, *Orai2* and *Shank2* exonic and 3'UTR sites (magenta bars) were selected for analysis in XEN cells. Additional negative control sites were selected from the *Dhcr7* gene, which is located 80 kb upstream of the *Osbpl5* gene. (**b**) NUP153, mAb414 and NUP98 ChIP were performed at NUP153 positive and negative sites in XEN cells. Of the three NUP153 positive sites, the *Vim*, *Orai2*, and *Shank2* promoters displayed NUP153 enrichment in XEN cells. mAb414 (NUP107/62) and NUP98 enrichment was also observed at the *Orai2* and *Shank2* promoters. **(c)** H3 ChIP at the *Vim*, *Orai2, Shank2* and *Dhcr7* promoter regions demonstrated equal allelic enrichment. (**d**) Allelic representation of input chromatin showing equal enrichment of both maternal and paternal alleles for target sites. (**e**) At the *Vim* promoter, NUP153 enrichment was observed at both parental alleles. At the *Orai2* promoter, NUP153, mAb414 and NUP98 bound to both the maternal and paternal alleles. At the *Shank2* promoter, NUP153, mAb414 and NUP98 were significant enriched at the paternal allele (n=3 biological samples with 3 technical replicates per sample). Error bars, s.e.m.; *, significance $p < 0.05$ compared to the siNT. Note: the same extracts used in Supplementary Figure 9 were used as Figure 3.

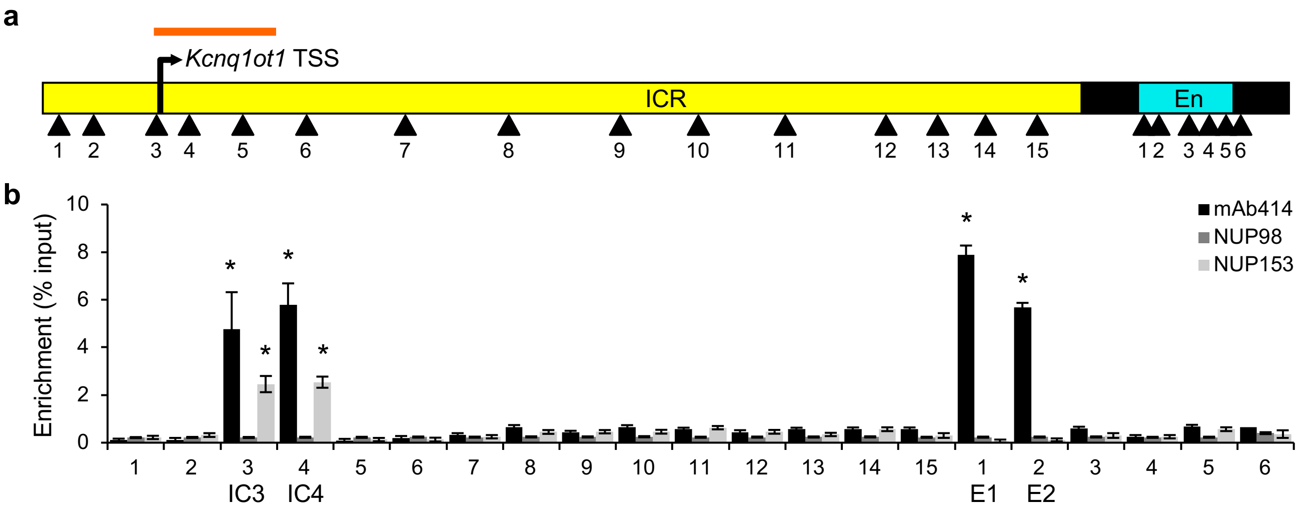

Supplementary Figure 10: mAb414, NUP98 and NUP153 enrichment at the *Kcnq1ot1* ICR and enhancer element in XEN cells.
(**a**) Twenty-one sites (arrowheads) across the *Kcnq1ot1* ICR and enhancer element were examined for mAb414, NUP98 and NUP153 enrichment. Orange bar represents mapped NUP153 binding site from ES cell DamID-seq data (Jacinto et. al, 2015). (**b**) Of the 21 sites, significant enrichment was observed for mA414 at regions IC3, IC4, E1 and E2, and for NUP153 at regions IC3 and IC4. No significant enrichment was found for NUP98 at any of the 21 sites. ICR, imprinting control region; En, enhancer element; TSS, transcription start site (n=3 biological samples with 3 technical replicates per sample). Error bars, s.e.m.; *, significance $p < 0.05$ compared to the IgG controls.

Sachani Supplementary Figure 11

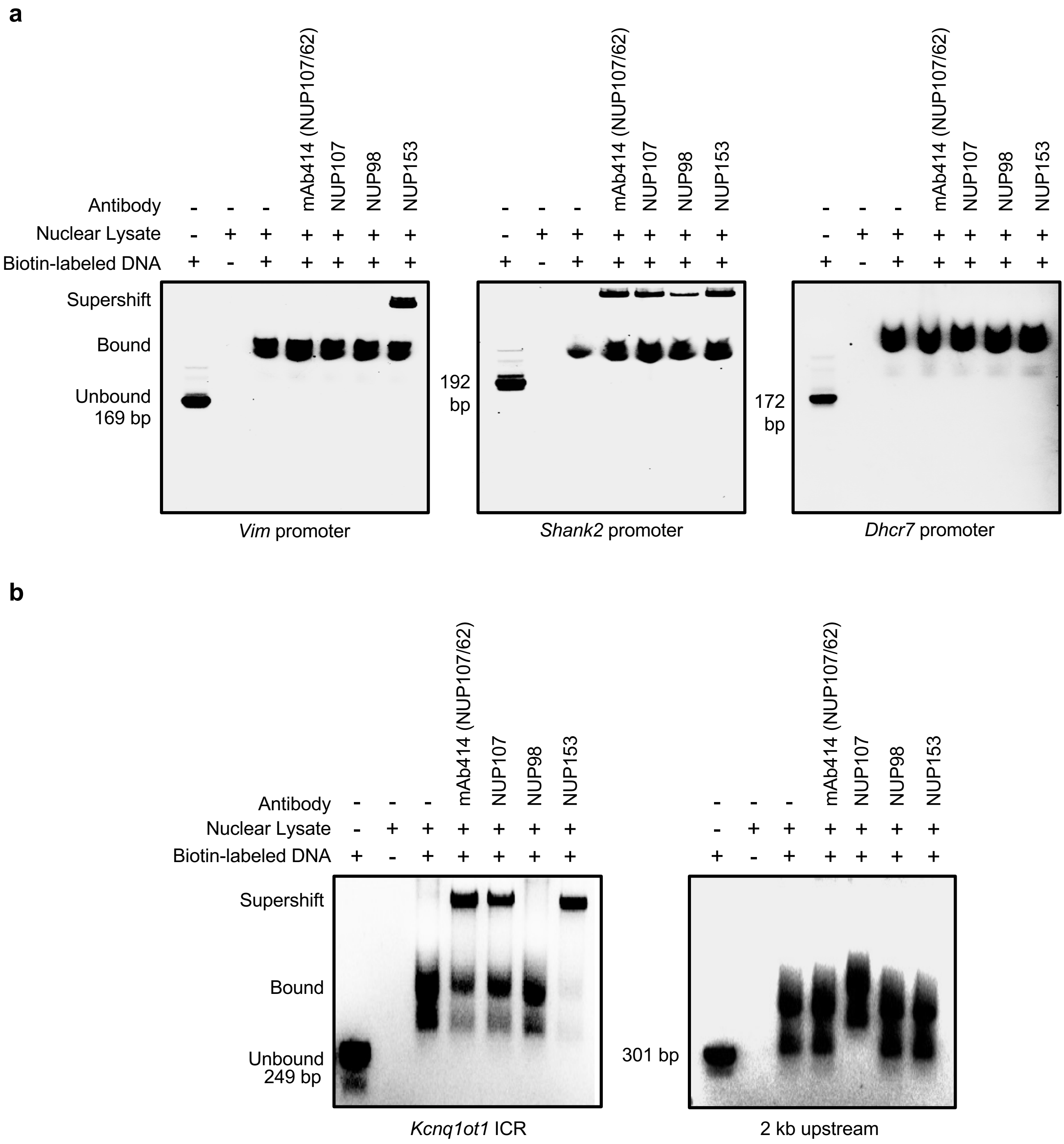

Supplementary Figure 11: Electromobility shift assays to validate NUP107, NUP62 and NUP153 binding to *Kcnq1ot1* ICR

(**a**) Prior to determining whether NUPs bind to *Kcnq1ot1* ICR, positive (*Vim* and *Shank2* promoter fragments) and negative (*Dhcr7* promoter fragment) sites for mAb414 (NUP107, NUP62), NUP98 and NUP153 enrichment (Supplementary Fig. 9) were validated by electromobility shift assay. Electromobility shift assays were performed using biotin-labeled probes along with mAb414, NUP107, NUP98 and NUP153 antibodies. Consistent with the ChIP data (Supplementary Fig. 9), the *Vim* promoter fragment was supershifted by NUP153 but not mAb414 (NUP107, NUP62), NUP107 or NUP98. The *Shank2* promoter fragment was supershifted by NUP153, mAb414 (NUP107, NUP62), NUP107, and NUP98. No supershift was observed for *Dhcr7* fragment with NUP153, mAb414 (NUP107, NUP62), NUP107 or NUP98 antibodies (n=2 biological samples, 2 replicates).

(b) NUP107, NUP62 and NUP153 supershifted a *Kcnq1ot1* ICR fragment containing the IC3 enrichment site but not a negative control DNA fragment located 2-kb upstream of the *Kcnq1ot1* ICR. Electromobility shift assays were performed using biotin-labeled probes along with NUP153, mAb414, NUP107, NUP98 and NUP153 antibodies. Supershifts were observed for the *Kcnq1ot1* ICR biotin-labelled probe and mAb414 (NUP62/107), NUP107 and NUP153 but not NUP98 antibodies and not for a biotin-labelled control region 2 kb upstream of the ICR (n=2 biological samples, 4 replicates). Note: the two extracts in (a) were two of the same extracts used in (b). Full blot images are shown.

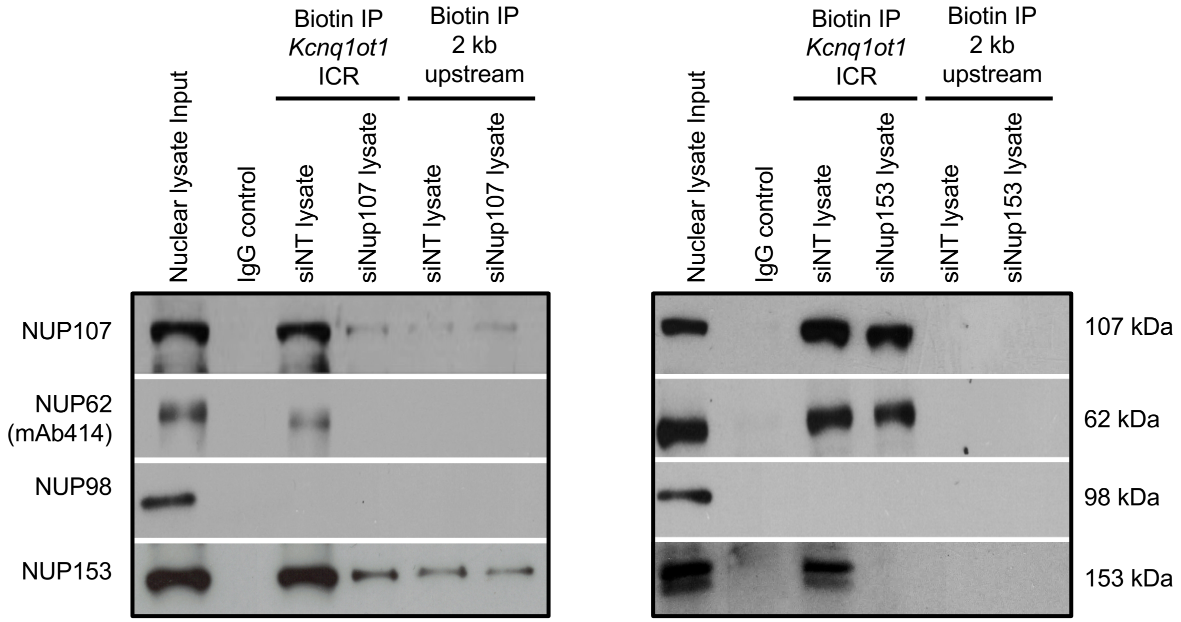

Supplementary Figure 12: NUP107, NUP62 and NUP153 bound to a *Kcnq1ot1* ICR fragment.

NUP107, NUP62 and NUP153 bound to a *Kcnq1ot1* ICR fragment but not to a control fragment. Biotin-labeled fragments were incubated with control and *Nup107-* and *Nup153*-depleted XEN cell lysates, and then were immunoprecipitated using a streptavidin antibody attached to paramagnetic beads. Following IP washes, samples were subjected to Western blot analysis using NUP107, mAb414 (NUP62), NUP98 and NUP153 antibodies (n=2 biological replicates). In addition to NUP107, NUP62 and NUP153 binding was reduced at the *Kcnq1ot1* ICR in *Nup107*-depleted samples, suggesting that NUP107 was required for NUP62 and NUP153 binding *in vitro. Nup153* depletion showed reduced NUP153 binding at the *Kcnq1ot1* ICR. However, NUP107 and NUP62 binding at the *Kcnq1ot1* ICR in *Nup153*-depleted samples was similar to that in the siNT control. By comparison, NUP98 did not bind the *Kcnq1ot1* ICR. Full blot images are shown in Supplementary Figure 33.

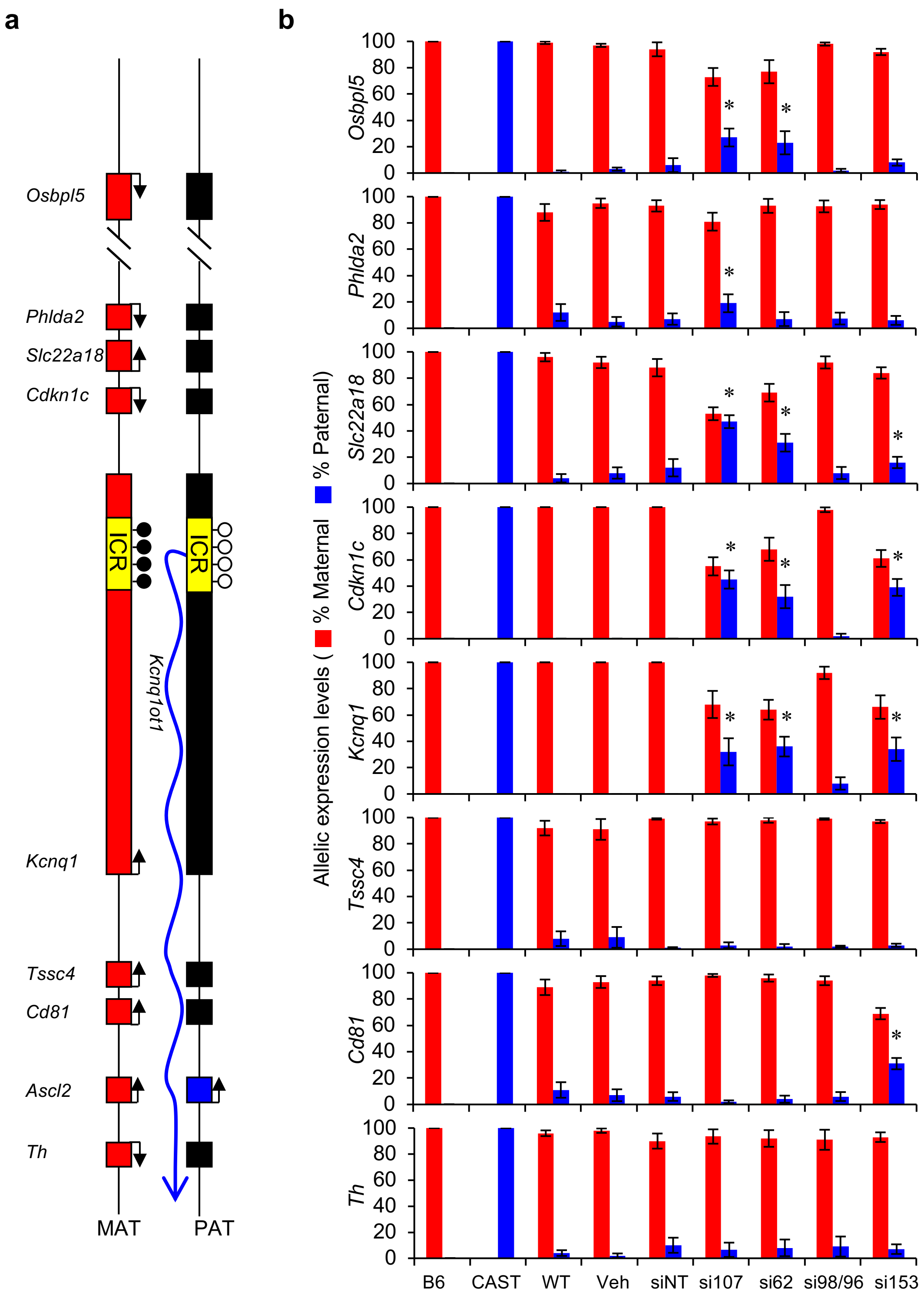

Supplementary Figure 13: Reactivation of a subset of paternal alleles at the *Kcnq1ot1* domain upon *Nup107, Nup62* and *Nup153* depletion using PCR and strain-specific restriction digestion.

(**a**) The *Kcnq1ot1* imprinted domain. (**b**) Allelic expression analysis of imprinted genes in control and *Nup*-depleted XEN cells (n=3 biological samples with 3-5 technical replicates per sample). Error bars, s.e.m.; *, significance $p < 0.05$ compared to the WT control.

Sachani Supplementary Figure 14

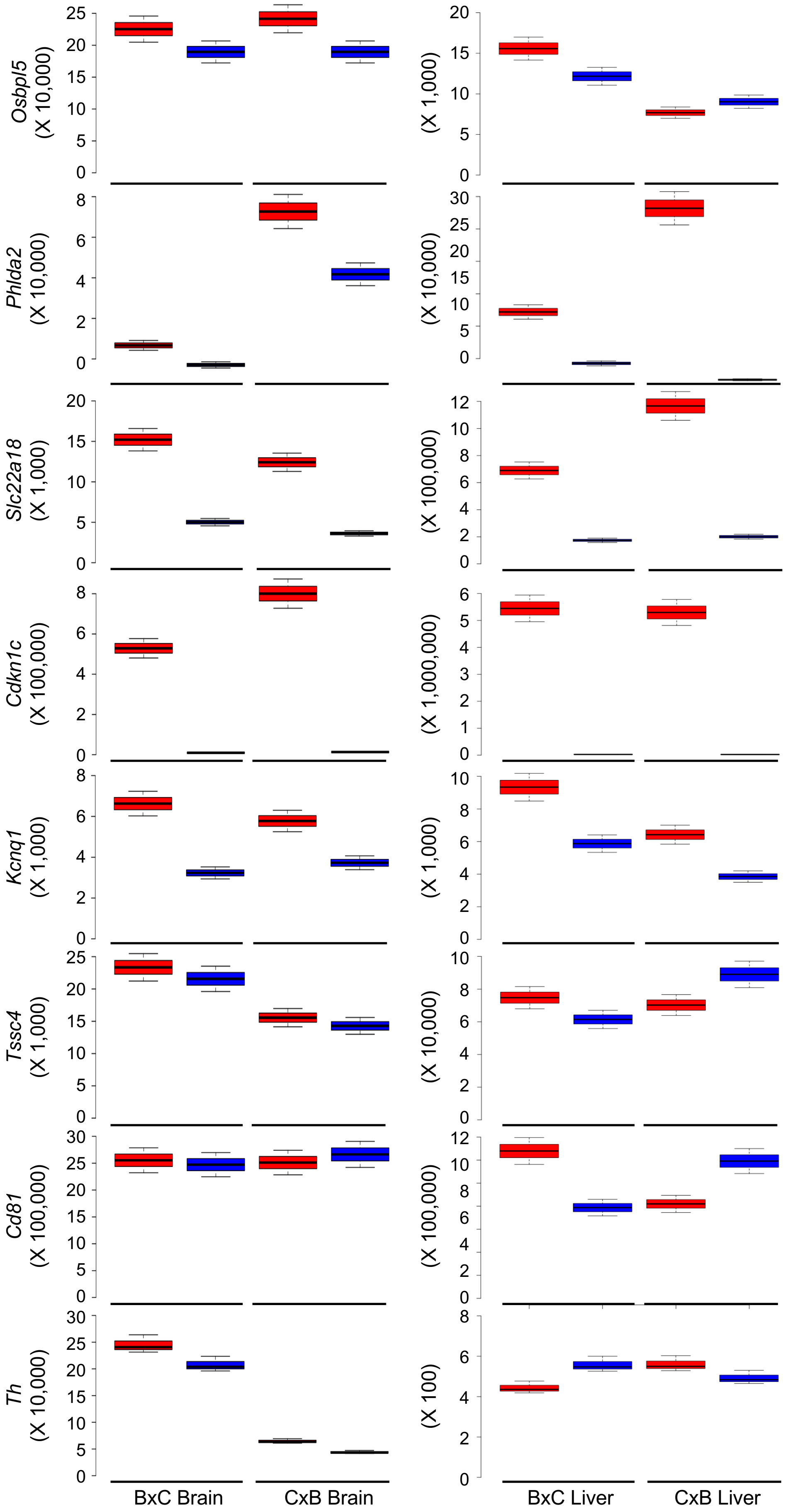

Supplementary Figure 14: Droplet digital PCR analysis of imprinted genes in the *Kcnq1ot1* domain in control reciprocal F_1 brain and liver tissues

(a) Probe specificity was determined using droplet digital PCR assays using stain-specific FAM and HEX probes on postnatal day 3 B6xCAST (BXC) brain, CXB brain, BxC liver and CxB liver (female listed first in crosses), and is represented as absolute quantification of B6 and CAST RNA copies in 1 μg. Notably, while there was variability in absolute transcript copies between tissues (higher in brain, *Osbpl5*, *Th*; higher in liver, *Slc22a18*, *Cdkn1c*, *Tssc4*; or ~equal, *Phlad2*, *Kcnq1*, *Cd81*), and between reciprocal crosses (*Phlda2*, *Th*), absolute transcript copies were highly similar in the 3 technical replicates, as well as in the pattern of parental expression (biallelic, *Osbpl5*, *Tssc4*, *Cd81*, *Th*; maternal bias, *Phlda2*, *Slc22a18*, *Kcnq1*; or maternal-specific, *Cdkn1c*). Center lines, medians; box limits, 25th and 75th percentiles as determined by R software; whiskers, 1.5 times the interquartile range from 25th and 75th percentiles.

Absolute allelic RNA copies μg^{-1}

		WT	Veh	siNT	si107	si62	si98/96	si153
Osbpl5	Maternal	61200	58963	58579	57225	53429	62875	58725
	Paternal	5454	5196	5558	18875	19025	6663	5450
Phlda2	Maternal	365	410	290	253	296	364	278
	Paternal	52	73	75	479	67	51	37
Slc22a18	Maternal	17500	27679	25600	18170	19117	19413	17742
	Paternal	4320	6075	6038	35025	37692	4346	37192
Cdkn1c	Maternal	1892	2333	2088	1871	1933	2175	1754
	Paternal	98	57	36	12742	10946	25	13217
Kcnq1	Maternal	260	243	260	302	297	265	308
	Paternal	47	53	34	850	779	60	773
Tssc4	Maternal	5129	4708	5125	5455	5621	5358	4963
	Paternal	1554	1494	1548	1658	1750	1623	1608
Cd81	Maternal	2267	2575	2083	2508	2441	2558	1883
	Paternal	36	52	62	51	32	55	13083
Th	Maternal	91	64	68	88	72	63	59
	Paternal	9	5	7	7	14	4	7

Supplementary Figure 15: Absolute allelic transcript abundance of imprinted genes in the Kcnq1ot1 imprinted domain as determined by ddPCR assays, corresponding to Figure 4.
Absolute quantification of B6 and CAST *Kcnq1ot1* RNA copies in 1 µg of RNA as detected by FAM and HEX probes in control and *Nup*-depleted XEN cells (n=3 biological samples) using droplet digital PCR.

Sachani Supplementary Figure 16

a

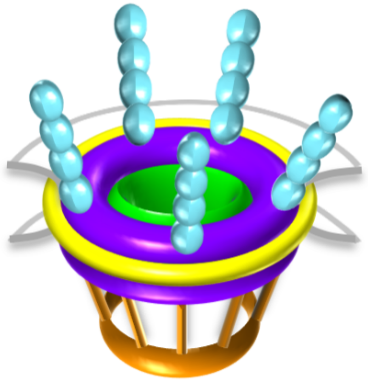

- Cytoplasmic filaments (NUP358)
- Nuclear and cytoplasmic rings (NUP107, NUP96, NUP93, NUP160, AHCTF1)
- Central channel filled with FG NUPs (NUP98, NUP62)
- Transmembrane ring
- Nuclear basket (NUP153, NUP50, TPR)

b

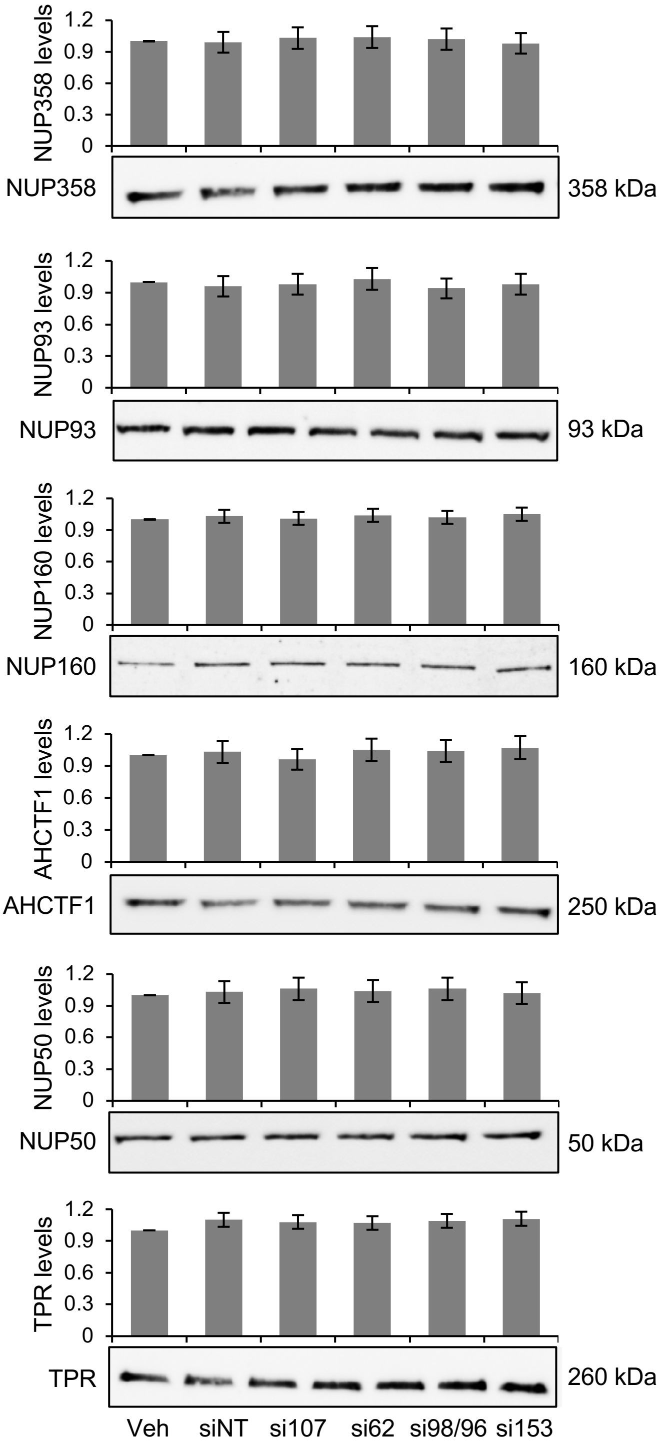

Supplementary Figure 16: Nucleoporin levels were not disrupted upon NUP107, NUP62, NUP98/96 or NUP153 depletion

(a) Schematic representation of nuclear pore complex.

(b) Nucleoporin levels were not altered upon *Nup107*, *Nup62, NUP98/96* and *Nup153* depletion. Western blot analysis using NUP50, NUP93, NUP160, NUP358, AHCTF1 (ELYS) and TPR antibodies was performed 48 hours after transfection. Nucleoporin levels were not significantly different between control and *Nup*-depleted XEN nuclear extracts. Tubulin was used as loading control. Error bars, s.e.m.; *, significance $p < 0.05$ compared to the Vehicle control; (n=3 biological samples). Full Western blot images are shown in Supplementary Figure 34.

Sachani Supplementary Figure 17

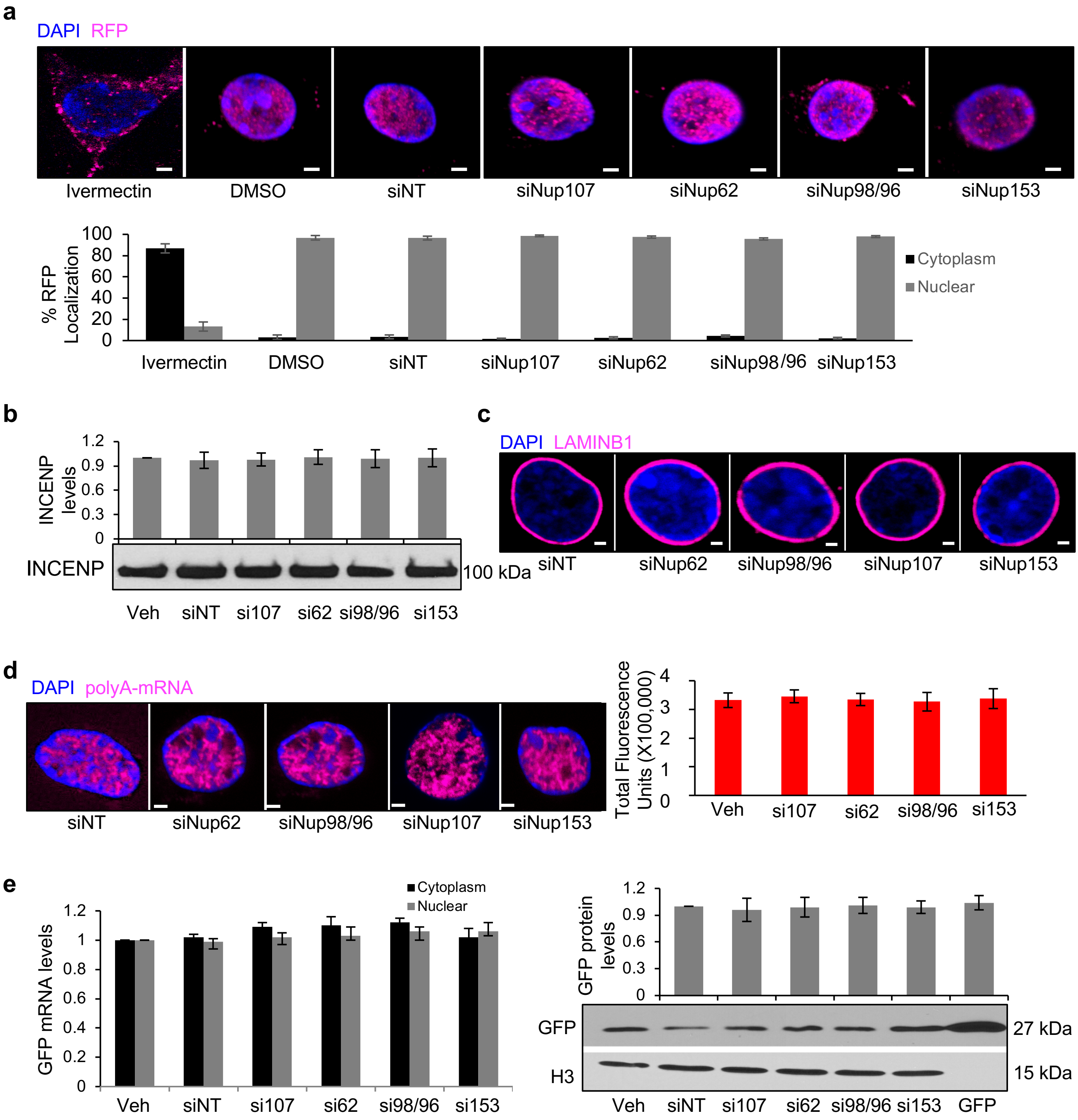

Supplementary Figure 17: Nuclear transport was not altered upon nucleoporin depletion.

(**a**) Endogenous E47-RFP^{NLS} protein transport was not disrupted upon nucleoporin depletion, compared to the ivermectin control where nuclear import was blocked in XEN cells. Percent nuclear E47-RFP^{NLS} localization was not significantly different between control and nucleoporin-depleted XEN cells, compared to ivermectin control. Scale bar, 1 μm; (n=60). In these images, red fluorescence was converted to magenta. (**b**) Endogenous protein transport was not disrupted upon nucleoporin depletion. INCENP protein levels were not significantly different between control and *Nup*-depleted XEN nuclear extracts, respectively. Histone 3 (H3) was used as a loading control (n=2-3 biological samples with 3 technical replicates per sample). (**c**) LAMINB1 protein transport was not altered upon nucleoporin depletion. Control and *Nup*-depleted XEN cells displayed LAMINB1 localization at the nuclear periphery. Scale bar, 1 μm; (n=30). In these images, red fluorescence was converted to magenta. (**d**) Nuclear polyA-mRNA retention levels were not changed upon nucleoporin depletion. Compared to controls, nucleoporin-depleted cells did not accumulate polyA-mRNA, showing no significant difference in biotin-labelled oligodT fluorescence levels corrected for background levels. Scale bar, 1 μm; error bars, s.e.m.; *, significance $p < 0.05$ compared to the siNT control; (n=30-45). In these images, red fluorescence was converted to magenta. (**e**) GFP mRNA and protein nuclear transport were not disrupted upon nucleoporin depletion. Nuclear and cytoplasmic GFP mRNA abundance relative to *Gapdh* expression and nuclear GFP protein levels using H3 as a loading control were similar in control and nucleoporin-depleted XEN cells. Error bars, s.e.m.; *, significance $p < 0.05$ compared to the siNT control; (n=3 biological samples with 3 technical replicates per sample). Full Western blot images are shown in Supplementary Figure 35.

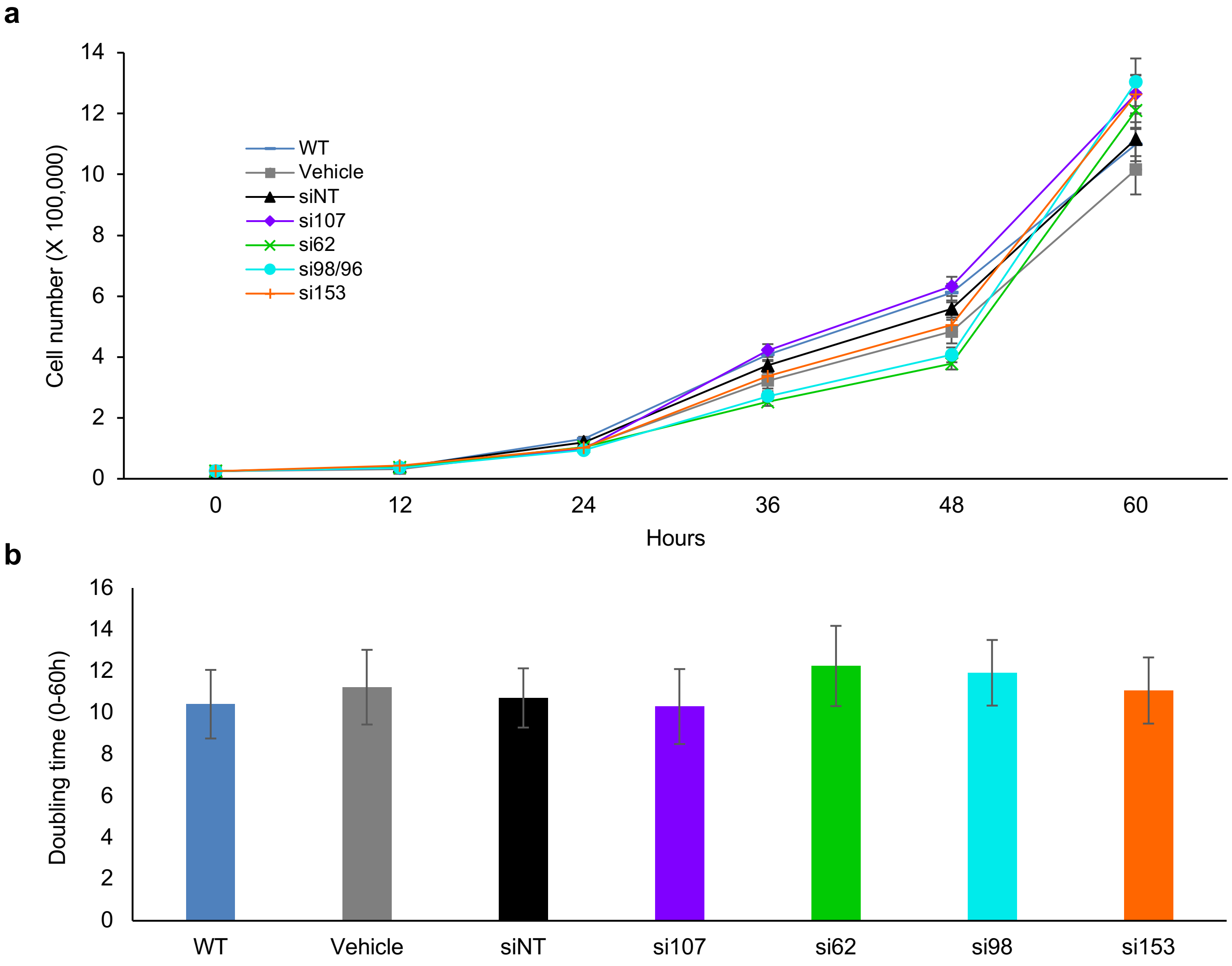

Supplementary Figure 18: XEN cell growth rate and doubling time was not altered upon nucleoporin depletion.

Approximately 25000 cells were seeded and then transfected 12 hours later with siRNAs. Control and *Nup*-depleted XEN cells were monitored for 60 hours. (**a**) Direct cell counts were performed every 12 hours (n=3 biological samples with technical replicates per sample). (**b**) Doubling time (DT) was calculated as specified in the ATCC guidelines using the formula DT = T * (ln (2) / ln (X_e/X_b)), T= time interval; X_e, Cell count at end of time interval; X_b, cell count at start of time interval. No significant change in cell growth rate or doubling time was observed at different time intervals between samples compared to the WT control. Error bars, s.e.m.

Sachani Supplementary Figure 19

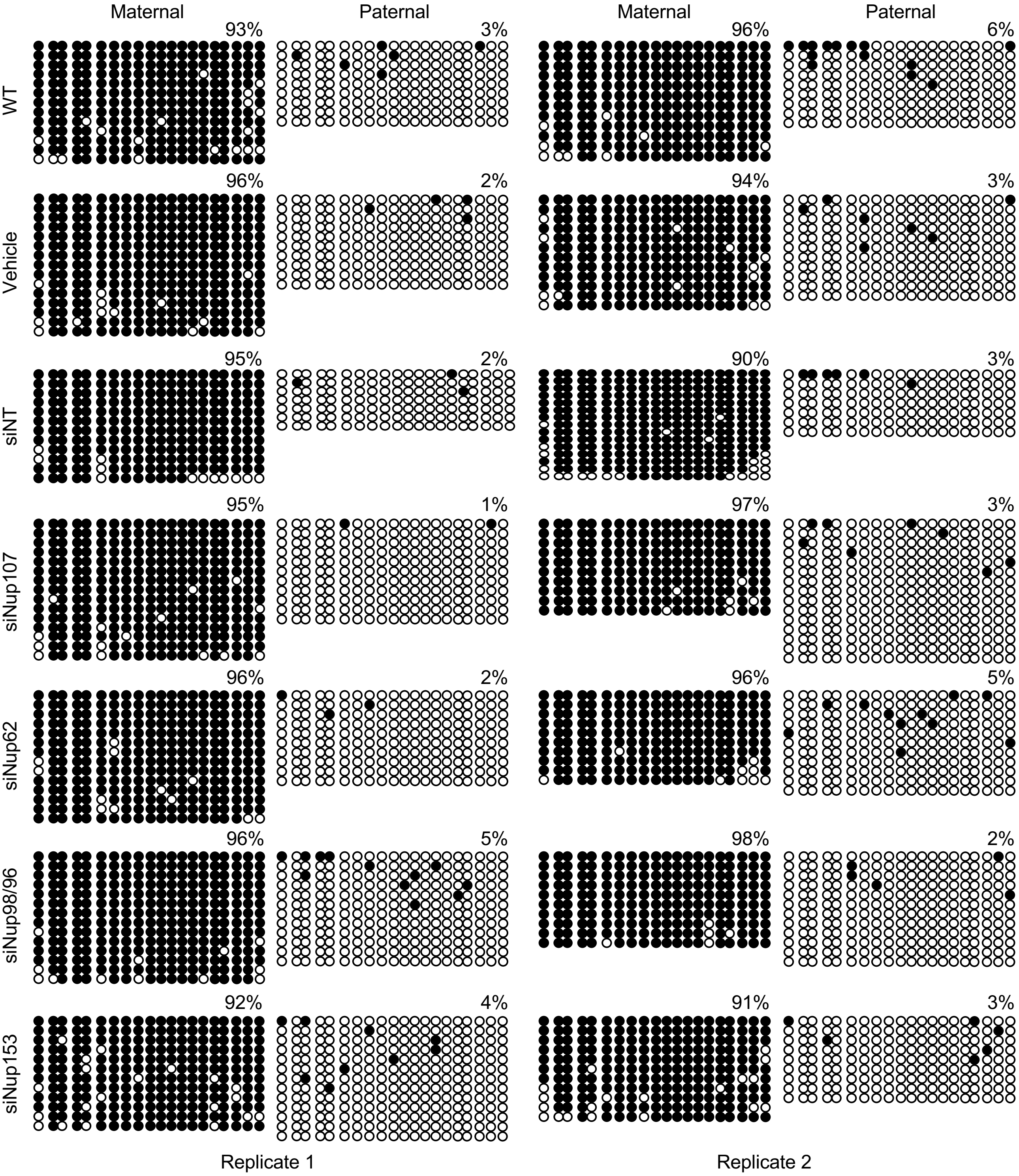

Supplementary Figure 19: DNA methylation was maintained upon nucleoporin depletion.

Methylation status of the *Kcnq1ot1* ICR in control and *Nup*-depleted XEN cells (n=2 biological samples). Black circles, methylated CpGs; white circles, unmethylated CpGs. Each line represents an individual DNA strand. Total methylation percent is represented above each set of DNA strands. WT, wildtype; Veh, vehicle; siNT, non-targeting siRNA; si107, *Nup107* siRNA; si62, *Nup62* siRNA; si98/96, *Nup98/96* siRNA; si153, *Nup153* siRNA.

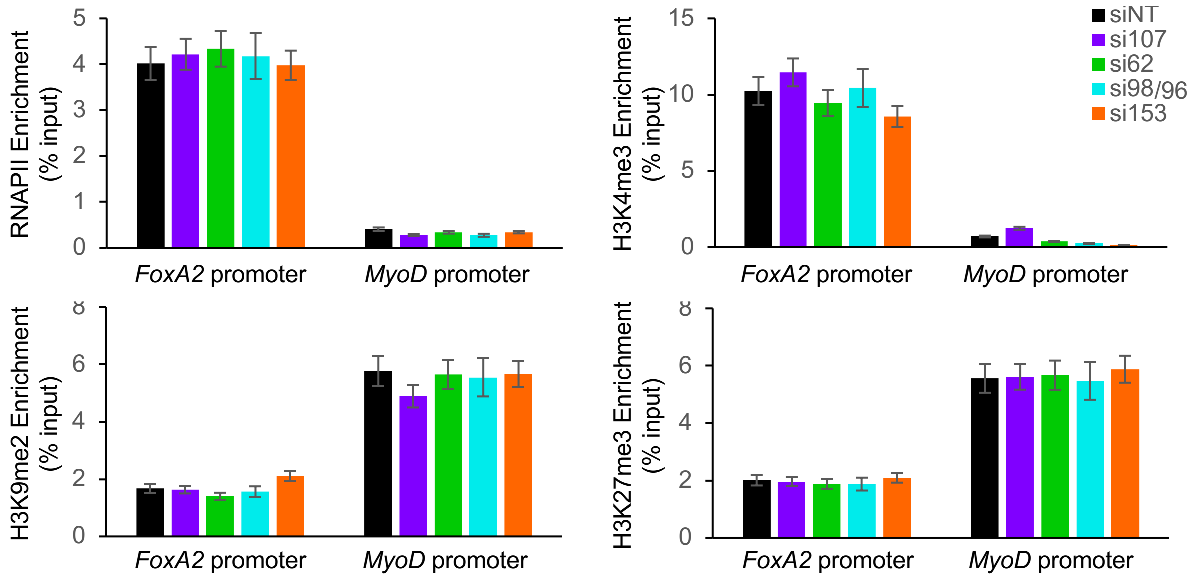

Supplementary Figure 20: Validation of active and repressive chromatin modifications at the *FoxA2* and *MyoD* genes.

From *Nup153* depletion RNA-seq data, Jacinto et al., 2015 observed no change in expression of pluripotency markers in ES cells, although repressed neural genes were reactivated. We selected a XEN-expressed marker, *Foxa2,* as well as XEN-repressed, muscle-specific gene, *MyoD*, as positive and negative controls (Lim et al. 2008; Golding et al. 2010). Active and repressive chromatin ChIP validation at the *FoxA2* and *MyoD* genes in XEN cells. ChIP analysis using RNAPII, H3K4me3, H3K9me2 and H3K27me3 antibodies at *FoxA2* and *MyoD* promoters. The expressed *FoxA2* promoter (Golding et al., 2010) harbored active chromatin modification, RNAPII and H3K4me3, and low levels of repressive modifications, H3K9me2 and H3K27me3. Conversely, the repressed *MyoD* promoter (Lim *et. al.* 2008) was enriched for repressive modifications but lacked active chromatin modification. No change in enrichment levels were observed upon nucleoporin depletion compared to the siNT control (n=3 biological samples with 3 technical replicates per sample). Error bars, s.e.m.; *, significance $p < 0.05$.

Sachani Supplementary Figure 21

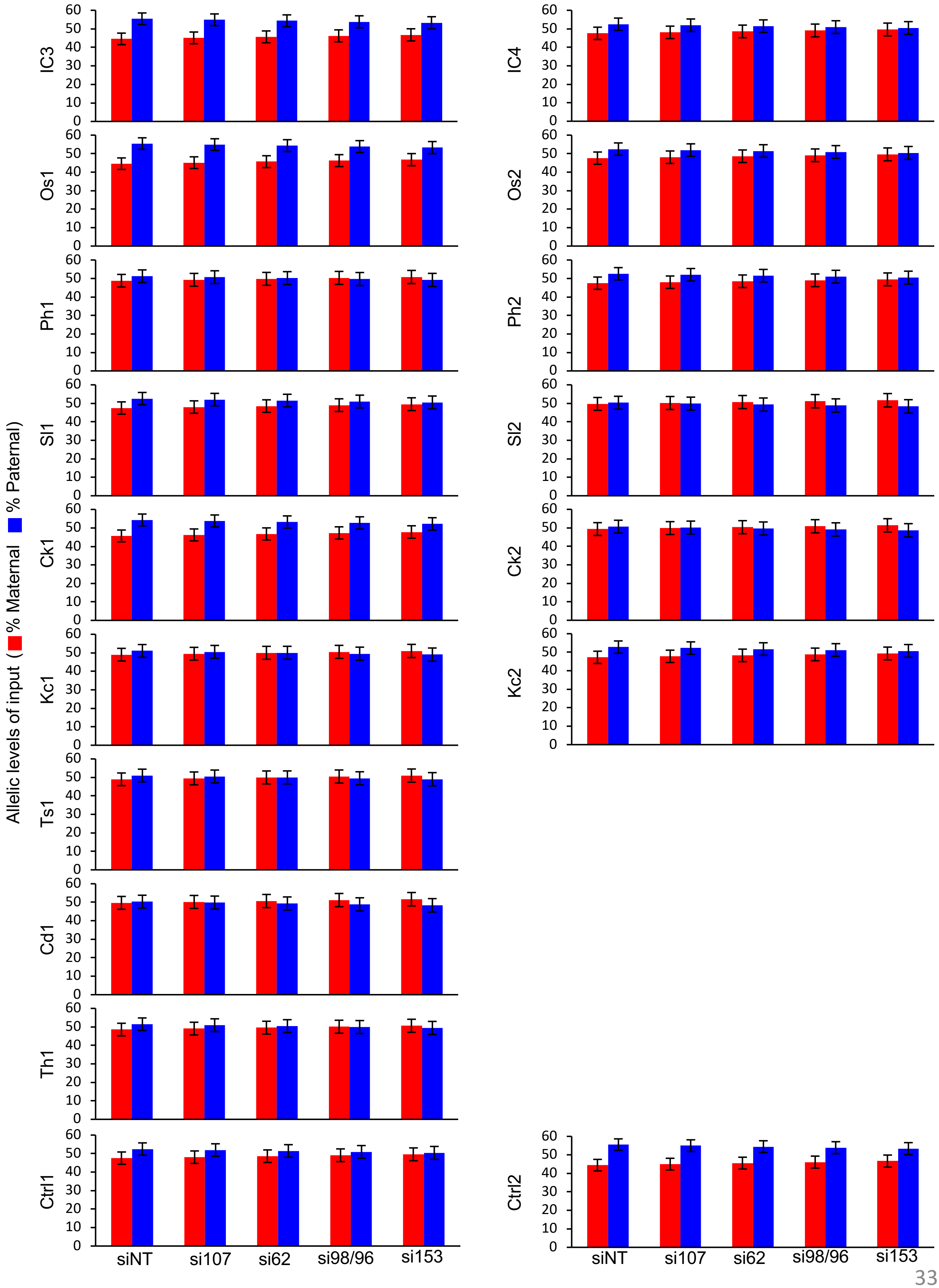

Supplementary Figure 21: Equal levels of the maternal and paternal alleles for regions assayed across the *Kcnq1ot1* imprinted domain from input chromatin.
Allelic analysis followed by quantification was performed for each primer pair on input chromatin. Error bars, s.e.m. Note: the same extracts used in Supplementary Figure 21 were used as Figure 3.

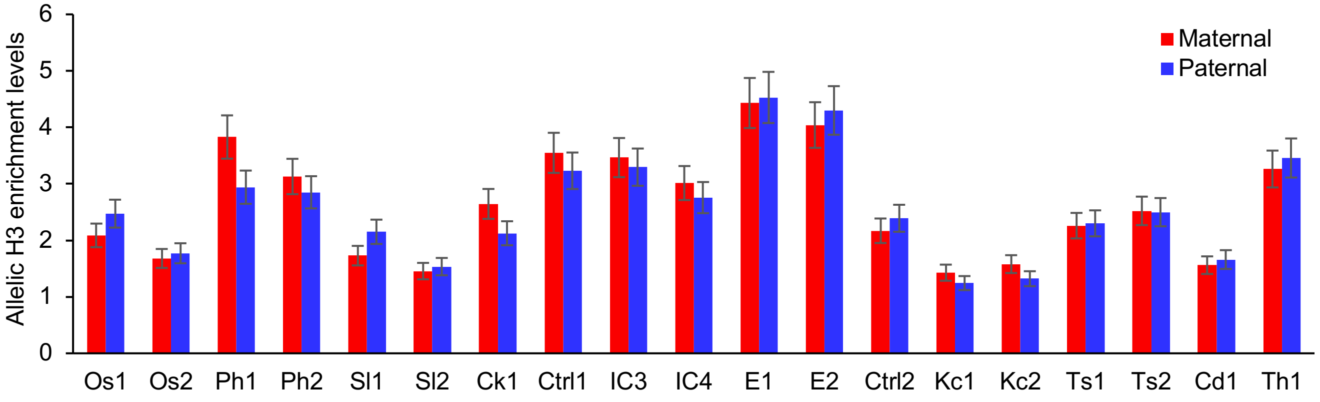

Supplementary Figure 22: Histone 3 was equally enriched on the maternal and paternal alleles for regions assayed across the *Kcnq1ot1* imprinted domain.
ChIP was performed at the *Kcnq1ot1* ICR, imprinted gene promoters and control regions using H3 antibodies. Allelic analysis followed by quantification was performed for each region on H3 enriched chromatin. No significant difference in allele-specific enrichment was observed. Error bars, s.e.m. Note: the same extracts used in Supplementary Figure 22 were used as Figure 3.

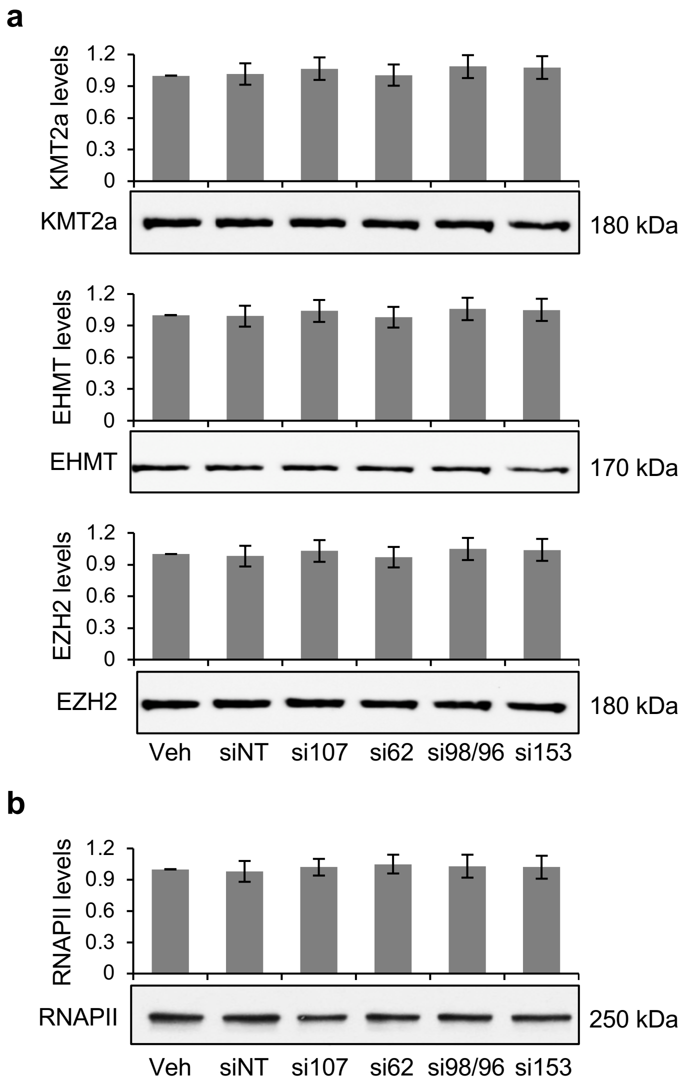

Supplementary Figure 23: Transcription regulatory protein levels were not disrupted upon *Nup107*, *Nup62*, *Nup98/96* or *Nup153* depletion
Transcription regulatory protein levels were not altered upon *Nup107*, *Nup62*, *Nup98/96* or *Nup153* depletion. Western blot analysis using **(a)** KMT2a, EHMT, and EZH2 or **(b)** RNAPII antibodies was performed 48 hours after transfection. No significant difference was observed between protein levels in control and *Nup*-depleted XEN nuclear extracts. Tubulin was used as loading control. Error bars, s.e.m.; *, significance $p < 0.05$ compared to the Vehicle control; (n=3 biological samples). Full Western blot images are shown in Supplementary Figure 36.

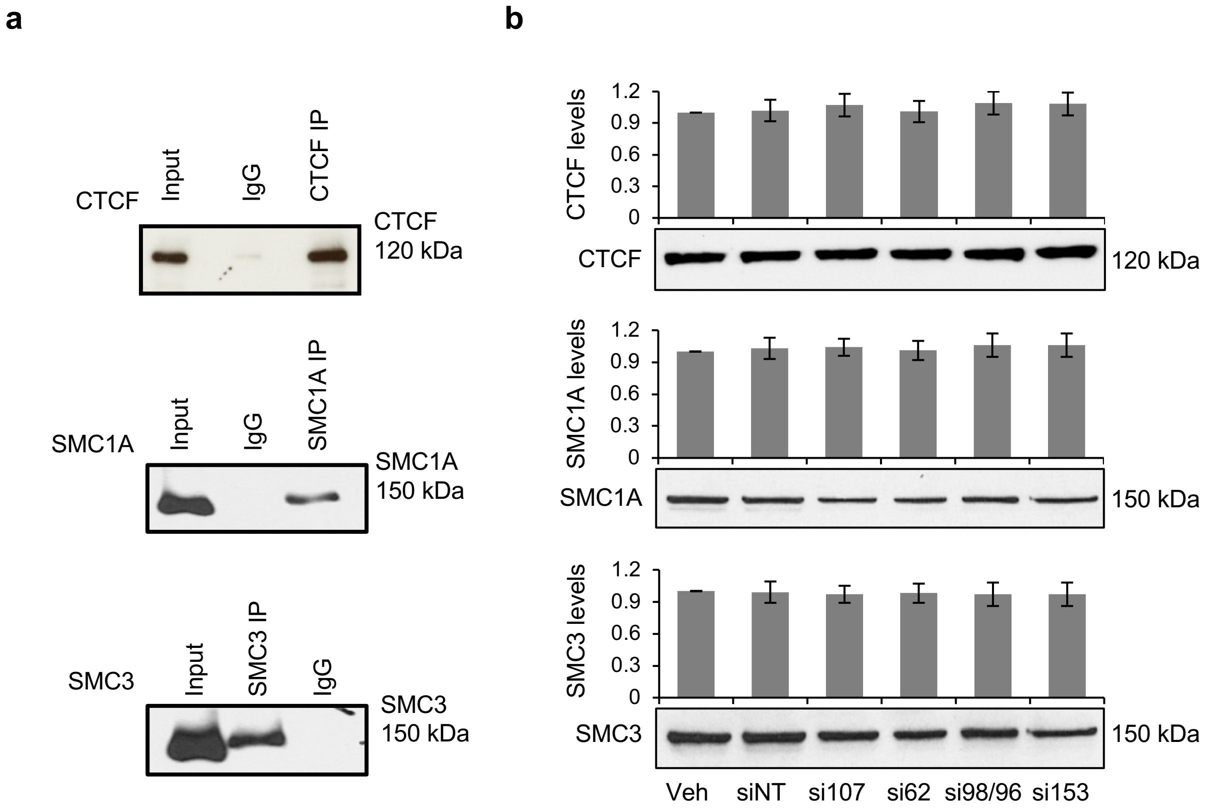

Supplementary Figure 24: CTCF, SMC1A and SMC3 antibody validation in XEN cells **(a)** CTCF, SMC1A and SMC3 immunoprecipitation was performed followed by Western blot analysis using the same antibody. Full blots are shown. (b) Western blot analysis using CTCF, SMC1A, and SMC3 antibodies was performed 48 hours after transfection. No significant difference was observed between CTCF, SMC1A, and SMC3 levels in control and *Nup*-depleted XEN nuclear extracts. Tubulin was used as loading control. Error bars, s.e.m.; *, significance $p < 0.05$ compared to the Vehicle control; (n=3 biological samples). Full Western blot images are shown in Supplementary Figure 37.

Sachani Supplementary Figure 25

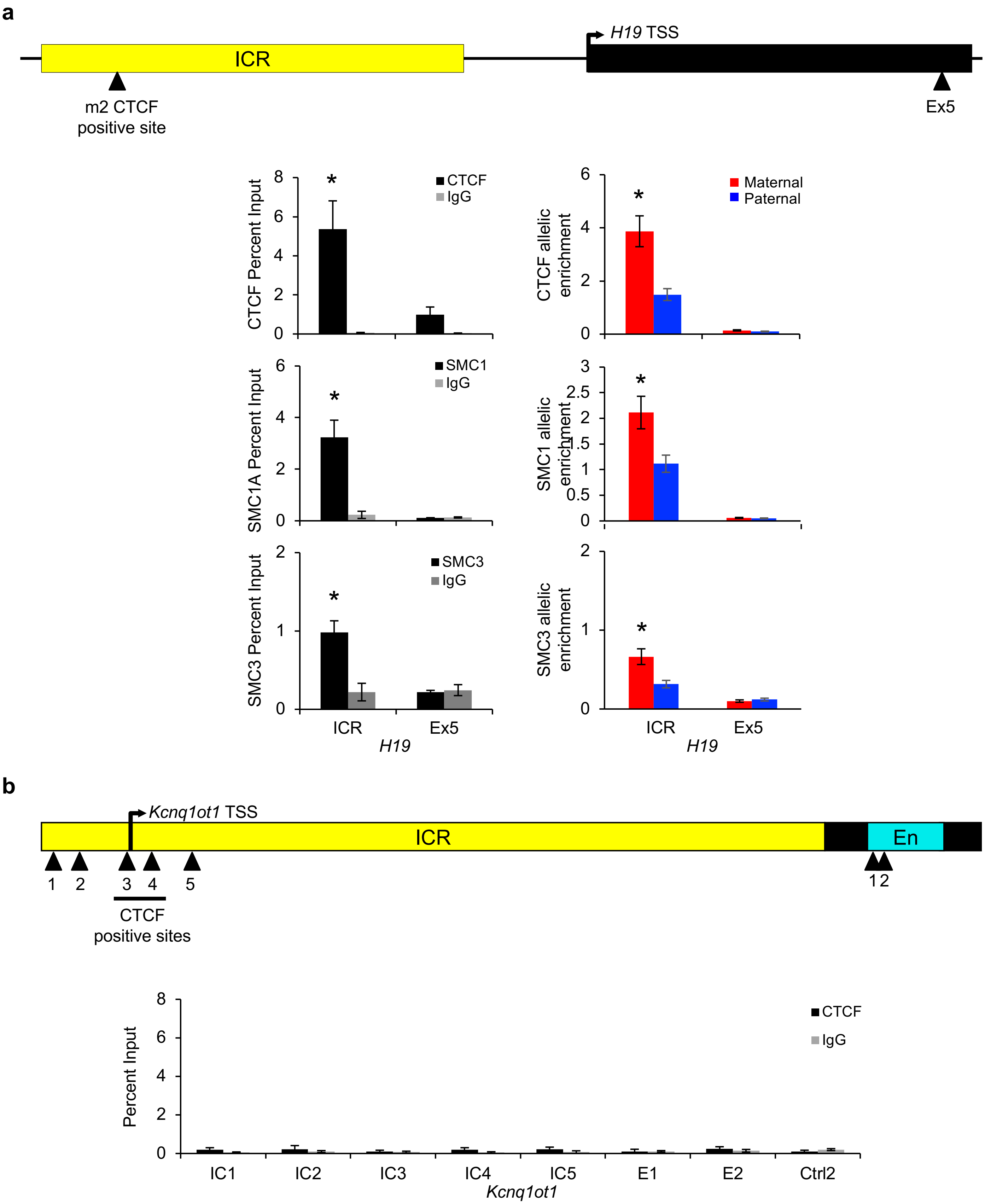

Supplementary Figure 25: CTCF, SMC1A and SMC3 enrichment at *H19* ICR in ES cells but not at *Kcnq1ot1* ICR in XEN cells.

(**a**) As a control for the CTCF, SMC1A, and SMC3 antibodies, one positive site at the *H19* ICR and one negative site within *H19* exon 5 (Ex5) (arrowheads) were examined for enrichment in ES cells. Significant CTCF, SMC1A, and SMC3 enrichment was observed at the maternal *H19* ICR as a positive control but not at the negative control exon 5 (Ex5) site (n=3 biological samples with 3 technical replicates per sample). (**d**) Seven sites (arrowheads) with mAb414 and/or NUP153 enrichment at the *Kcnq1ot1* ICR and enhancer element were examined for CTCF enrichment. No significant CTCF enrichment was observed at the *Kcnq1ot1* ICR and enhancer element in XEN cells (n=3 biological samples with 3 technical replicates per sample). Error bars, s.e.m.; *, significance $p < 0.05$ compared to IgG control.

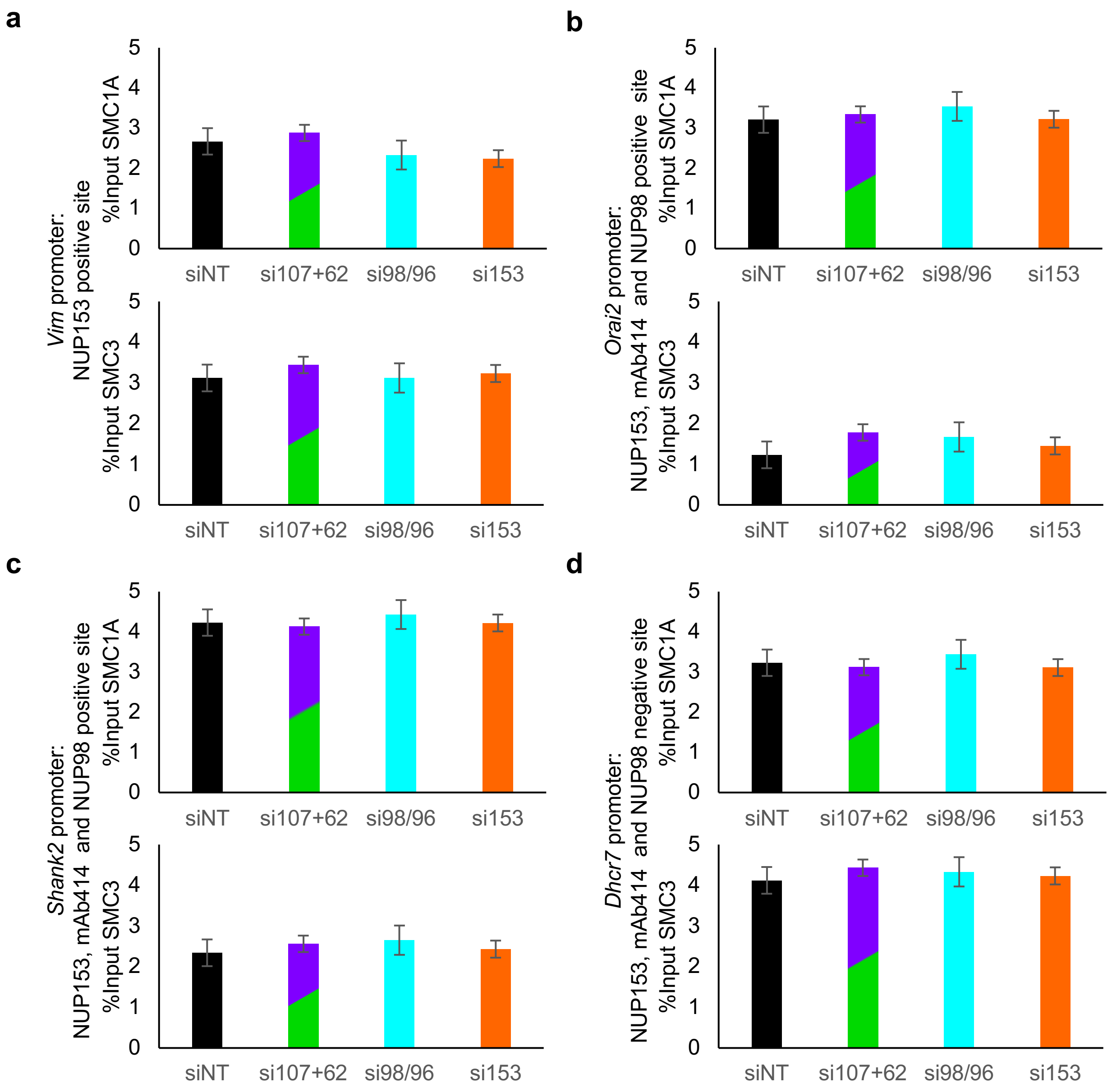

Supplementary Figure 26: SMC1A and SMC3 recruitment to the *Kcnq1ot1* ICR is nucleoporin-dependent

To determine whether SMC1A and SMC3 recruitment at other loci was dependent upon NUP107, NUP62, NUP98 or NUP153, ChIP was performed at previously identified NUP153, mAb414-, NUP98-binding positive sites, (**a**) *Vim*, (**b**) *Orai2*, and (**c**) *Shank2* promoters, and as well as a negative binding site, (**d**) *Dhcr7* promoter (Supplementary Figure 9) using SMC1A and SMC3 antibodies in control and *Nup*-depleted XEN cells. No significant change in SMC1A and SMC3 enrichment was observed at any of the sites tested, indicating that SMC1A and SMC3 recruitment to the *Kcnq1ot1* ICR was nucleoporin dependent (n=3 biological samples with 3 technical replicates per sample). Error bars, s.e.m.; *, significance $p < 0.05$ compared to siNT control. Note: Different extracts were used in Supplementary Figure 26 compared to Supplementary Figure 9 and Figure 3.

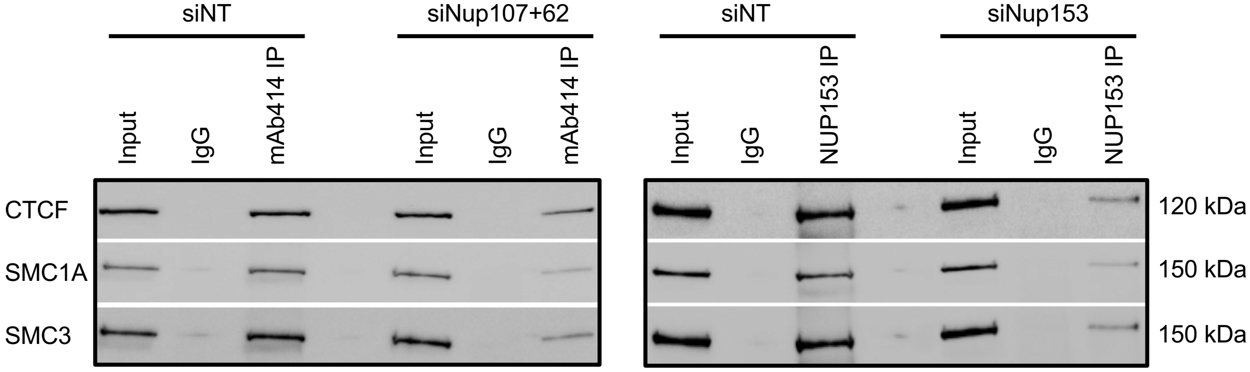

Supplementary Figure 27: While chromatin architectural protein levels were not disrupted upon *Nup107*, *Nup62*, *Nup98/96* or *Nup153* depletion, NUP107/62 and NUP153 interacted with CTCF and cohesin proteins

To validate nucleoporin-CTCF, and nucleoporin-cohesin interactions, NUP-immunoprecipitation followed by CTCF and cohesin protein Western blotting analysis was performed in control and *Nup107/62*- and *Nup153*-depleted XEN cells. In control cells, mAb414 and NUP153 were bound to CTCF, SMC1A, and SMC3. Upon *Nup107/62* and *Nup153* depletion, CTCF, SMC1A, and SMC3 interactions were reduced, indicating interactions between NUP107 and/or NUP62, and NUP153 and CTCF, SMC1A, and SMC3 (n=3 biological replicates). Full Western blot images are shown in Supplementary Figure 37.

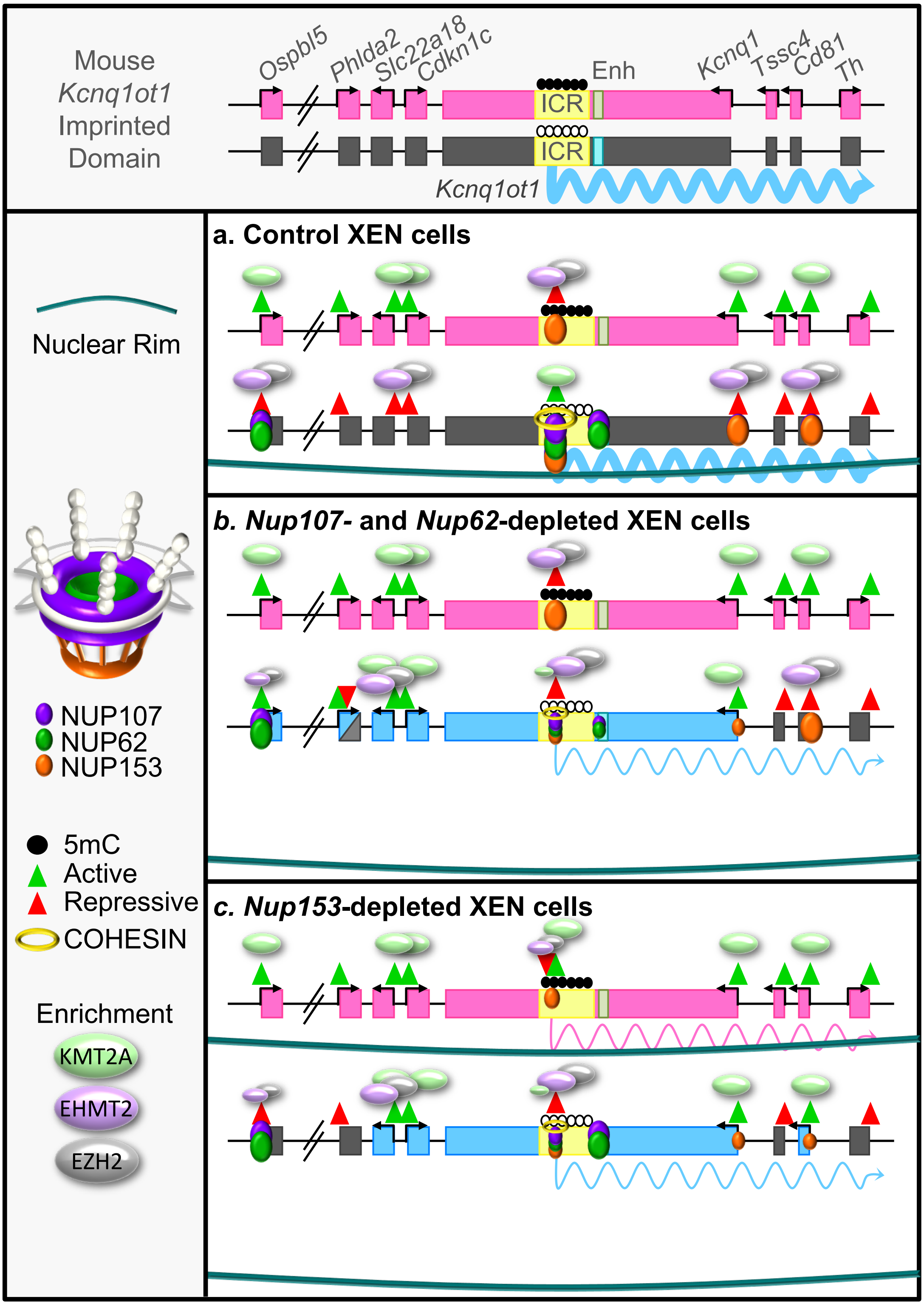
Mouse
Kcnq1ot1
Imprinted
Domain
Osbpl5
Phlda2
Slc22a18
Cdkn1c
Enh
Kcnq1
Tssc4
Cd81
Th
ICR
ICR
Kcnq1ot1
Nuclear Rim
NUP107
NUP62
NUP153
5mC
Active
Repressive
COHESIN
Enrichment
KMT2A
EHMT2
EZH2
a. Control XEN cells
b. Nup107- and Nup62-depleted XEN cells
c. Nup153-depleted XEN cells

Supplementary Figure 28: Summary of nucleoporin-mediated regulation of the *Kcnq1ot1* imprinted domain.

(**a**) Our data indicate that NUP107, NUP62 and NUP153 regulated paternal *Kcnq1ot1* ncRNA expression, paternal *Kcnq1ot1* domain positioning at the nuclear rim, and paternal allelic silencing of specific imprinted genes in XEN stem cells. NUP107, NUP62 and NUP153 were bound at the *Kcnq1ot1* ICR on the paternal allele in XEN cells. In addition, we found that NUP107, NUP62 and NUP153 maintained active chromatin at the *Kcnq1ot1* ICR. The cohesion complex, but not CTCF, assembled at the same sites within the paternal *Kcnq1ot1* ICR as NUP107, NUP62 and NUP153. KMT2A localized at sites with active chromatin, while EHMT2 and EZH2 localized at sites associated with repressive chromatin (*Tssc4* and *Th* not assayed), except at *Slc22a18*, where there was only KMT2a occupancy. (**b**) Upon *Nup107* and *Nup62* depletion, *Kcnq1ot1* ncRNA expression was significantly reduced, which was correlated with a change in histone modifications at the *Kcnq1ot1* ICR, and the paternal *Kcnq1ot1* domain was shifted away from the nuclear rim. NUP107 and NUP62 had reduced binding at the the *Kcnq1ot1* ICR and the enhancer element. NUP153 and cohesin binding were also reduced at the *Kcnq1ot1* ICR. Furthermore, the paternal allele of the core group of genes, *Slc22a18*, *Cdkn1c* and *Kcnq1* was reactivated, with a corresponding change in histone modifications and KMT2A, EHMT2 and EZH2 enrichment. The silent paternal *Osbpl5* and *Phlda2* were also reactivated with a corresponding change in histone modifications and KMTA, EHMT2 and EZH2 occupancy. (**c**) Similar alterations to *Nup107*- and *Nup62*-depleted XEN cells were observed upon *Nup153* depletion. Here, the normally-silent, maternal *Kcnq1ot1* ncRNA was reactivated. NUP153 had reduced binding at the paternal *Kcnq1ot1* ICR, *Kcnq1ot* promoter and *Cd81* promoter. NUP107, NUP62 and cohesin binding were also reduced at the *Kcnq1ot1* ICR. Furthermore, the paternal allele of the core group of genes, *Slc22a18*, *Cdkn1c* and *Kcnq1,* as well as *Cd81* were reactivated, with a corresponding change in histone modifications and KMT2A, EHMT2 and EZH2 enrichment. For all *Nup* depletions, maternal and paternal DNA methylation states were maintained.

NeoR

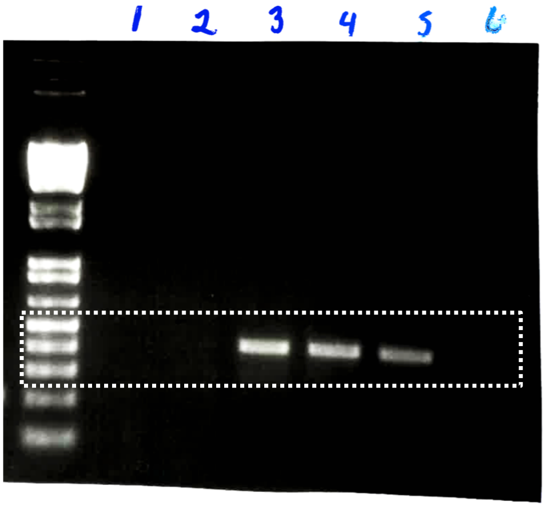

Mrpl

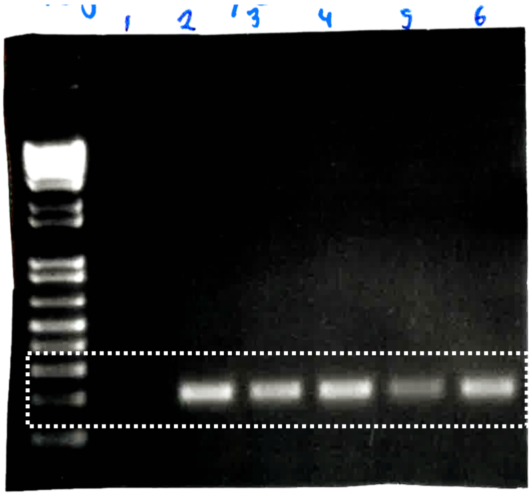

Supplementary Figure 29: Uncropped gel images for Supplementary Figure 2b.

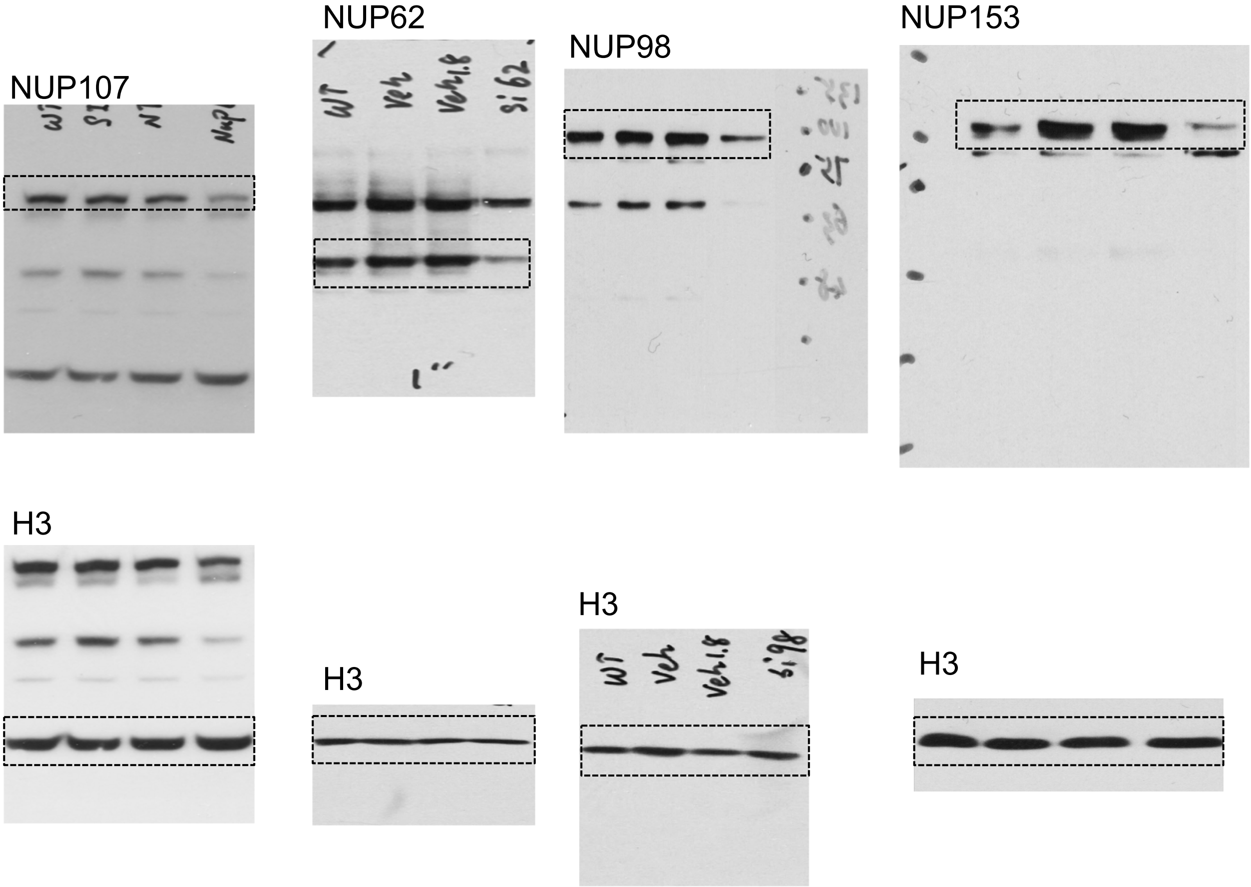

Supplementary Figure 30: Uncropped Western blot images for Supplementary Figure 4b.

Sachani Supplementary Figure 31

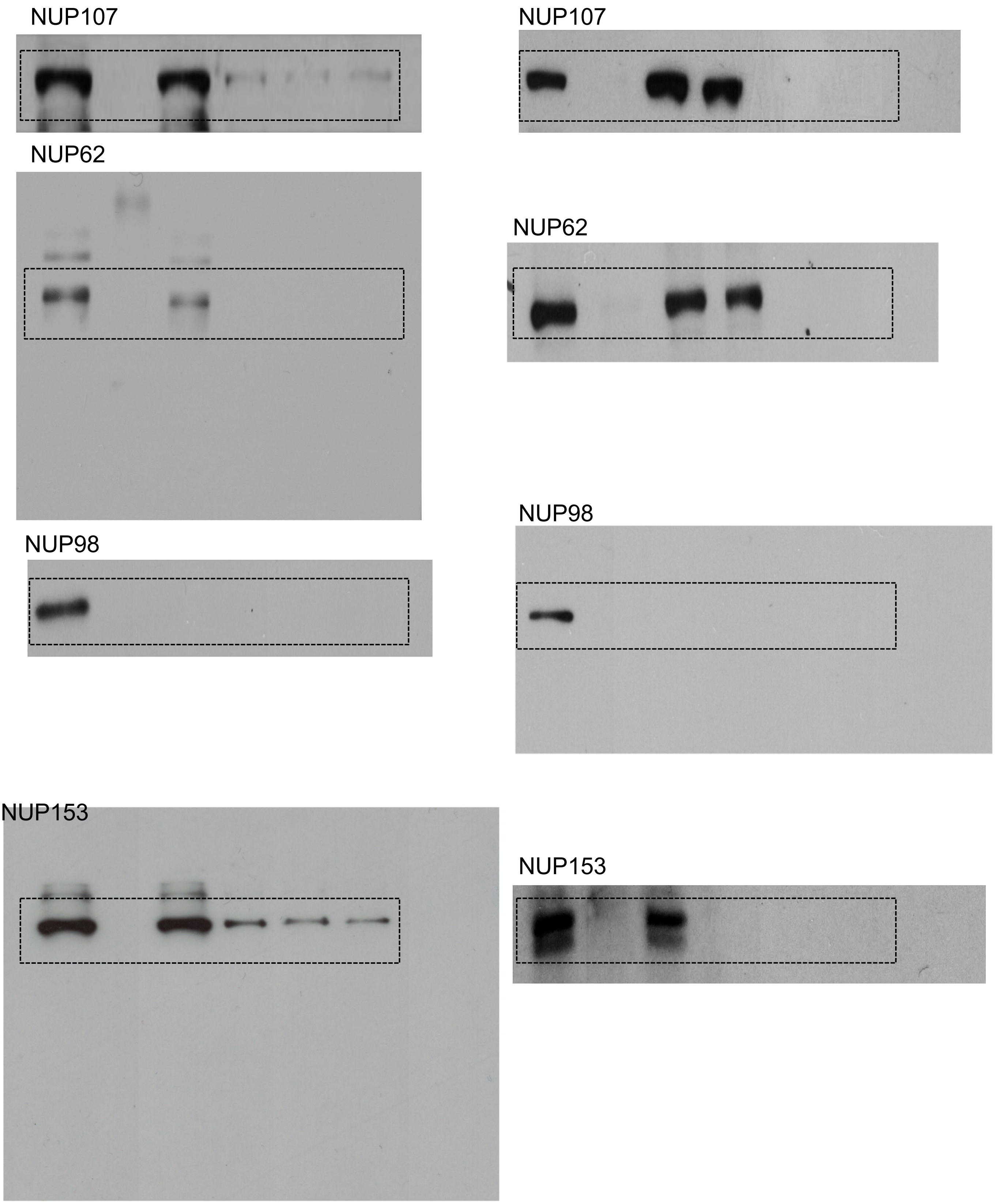

Supplementary Figure 31: Uncropped Western blot images for Supplementary Figure 12.

NUP358

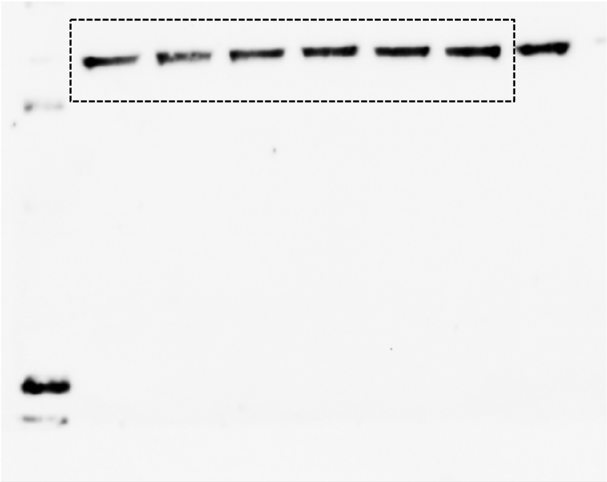

AHCTF1

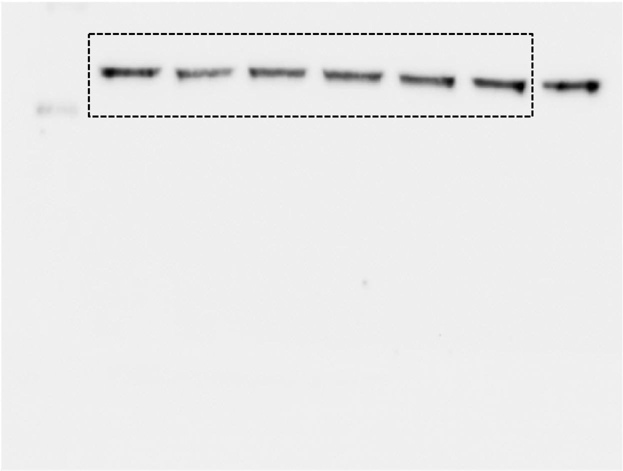

NUP93

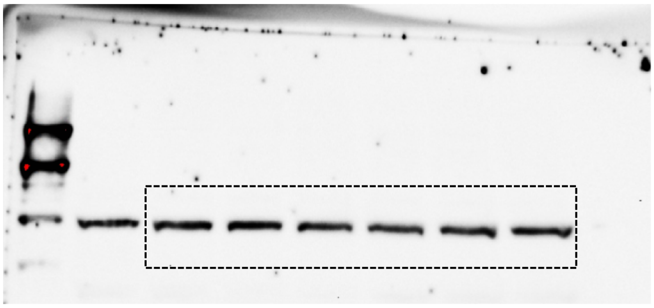

NUP50

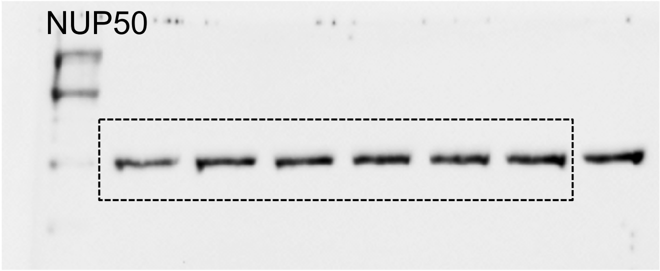

NUP160

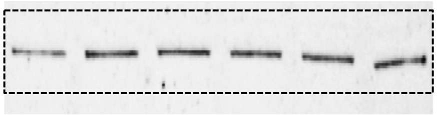

TPR

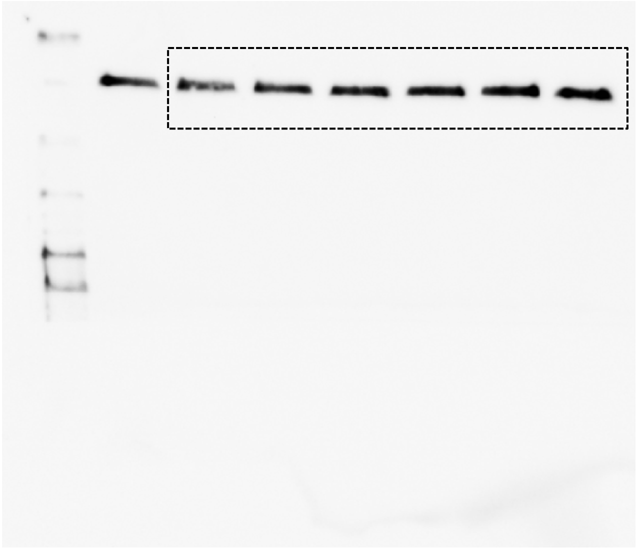

Supplementary Figure 32: Uncropped Western blot images for Supplementary Figure 16b.

INCENP

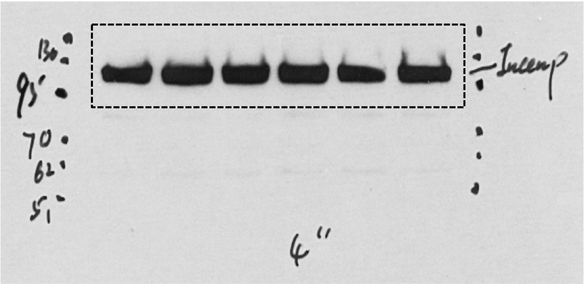

GFP

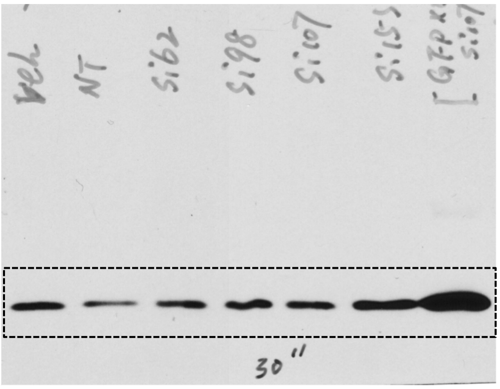

H3

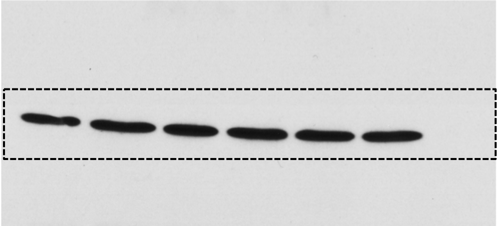

Supplementary Figure 33: Uncropped Western blot images for Supplementary Figure 17b,e.

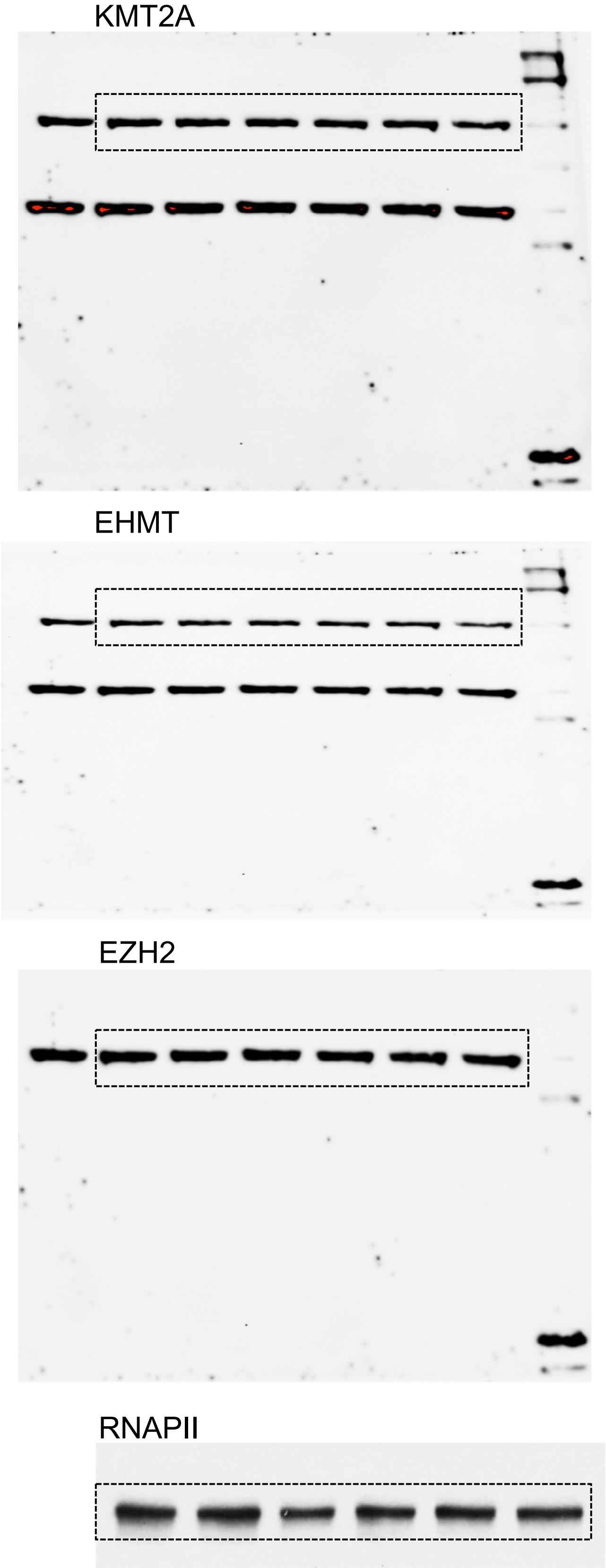

Supplementary Figure 34: Uncropped Western blot images for Supplementary Figure 23a,b.

CTCF

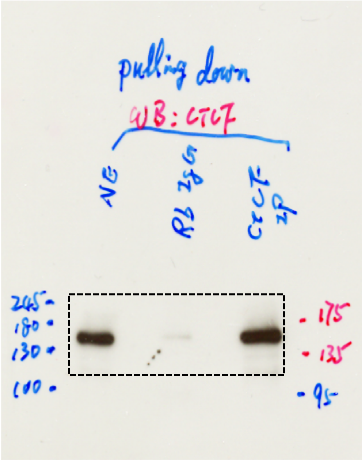

CTCF

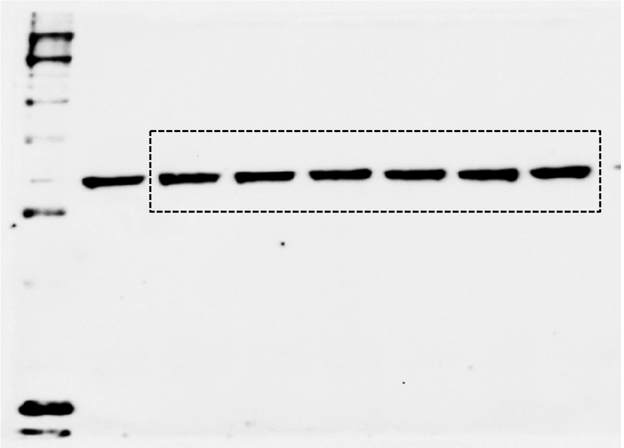

SMC1A

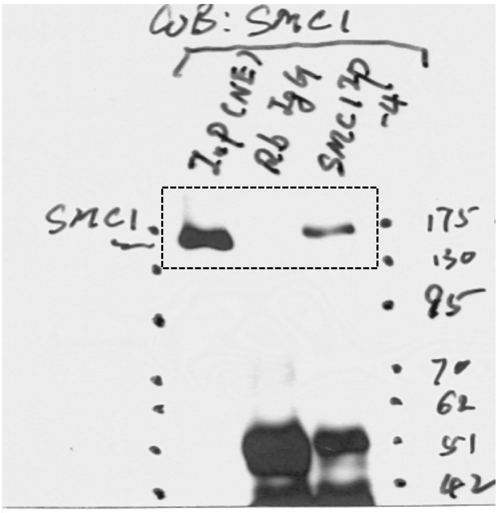

SMC1A

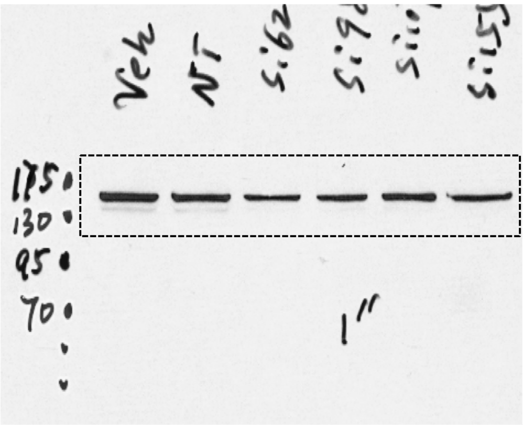

SMC3

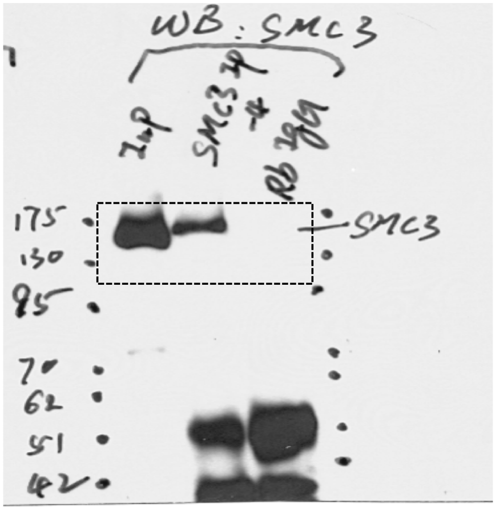

SMC3

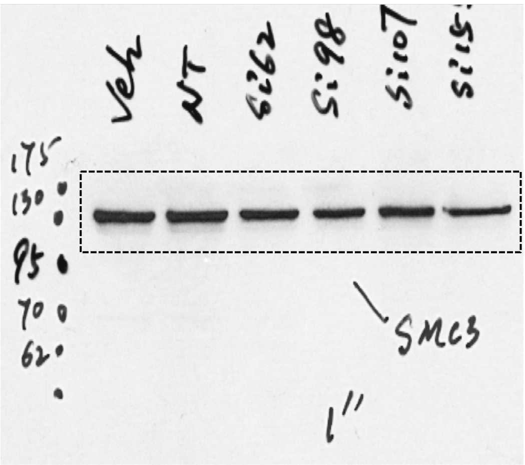

Supplementary Figure 35: Uncropped Western blot images for Supplementary Figure 24a,b.

CTCF

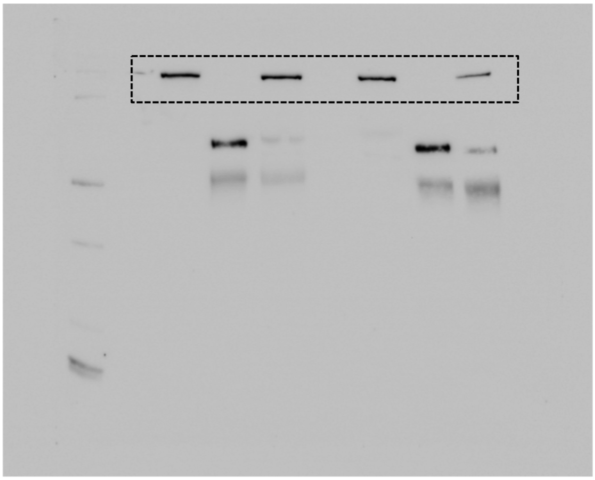

CTCF

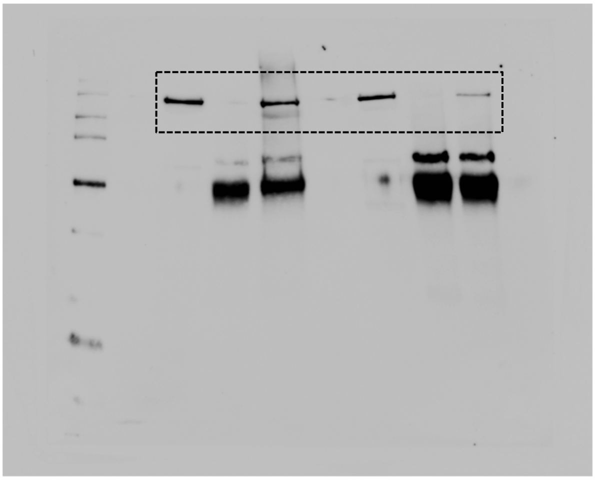

SMC1A

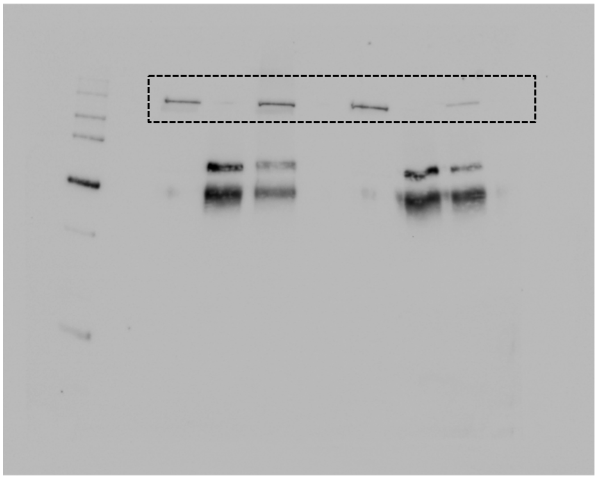

SMC1A

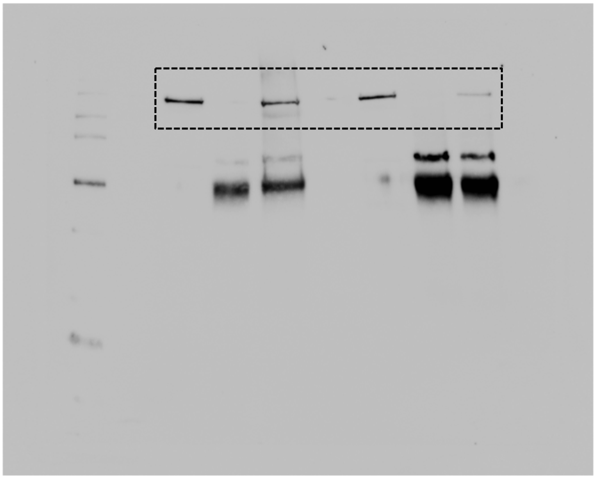

SMC3

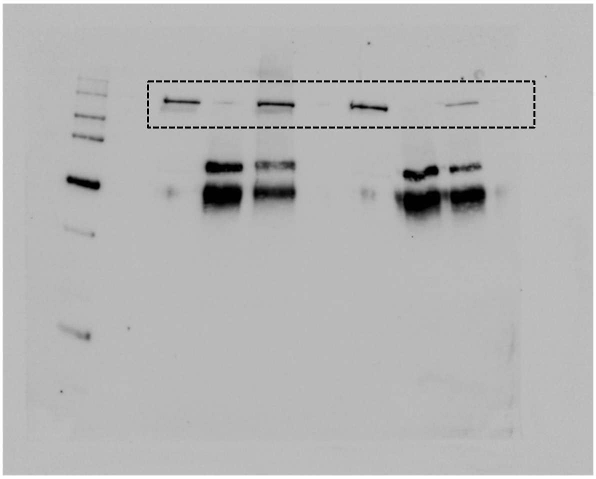

SMC3

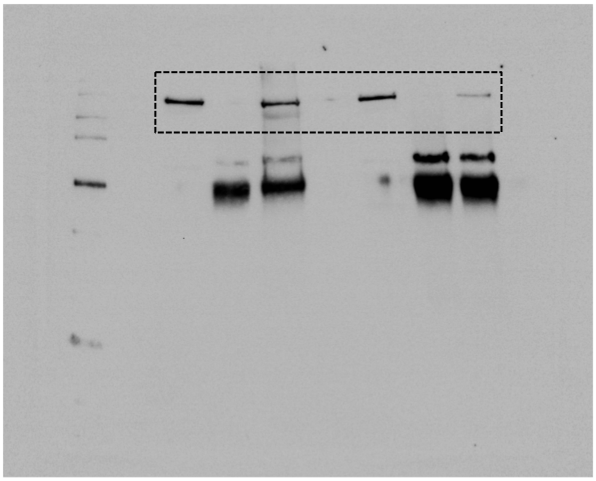

Supplementary Figure 36: Uncropped Western blot images for Supplementary Figure 27.

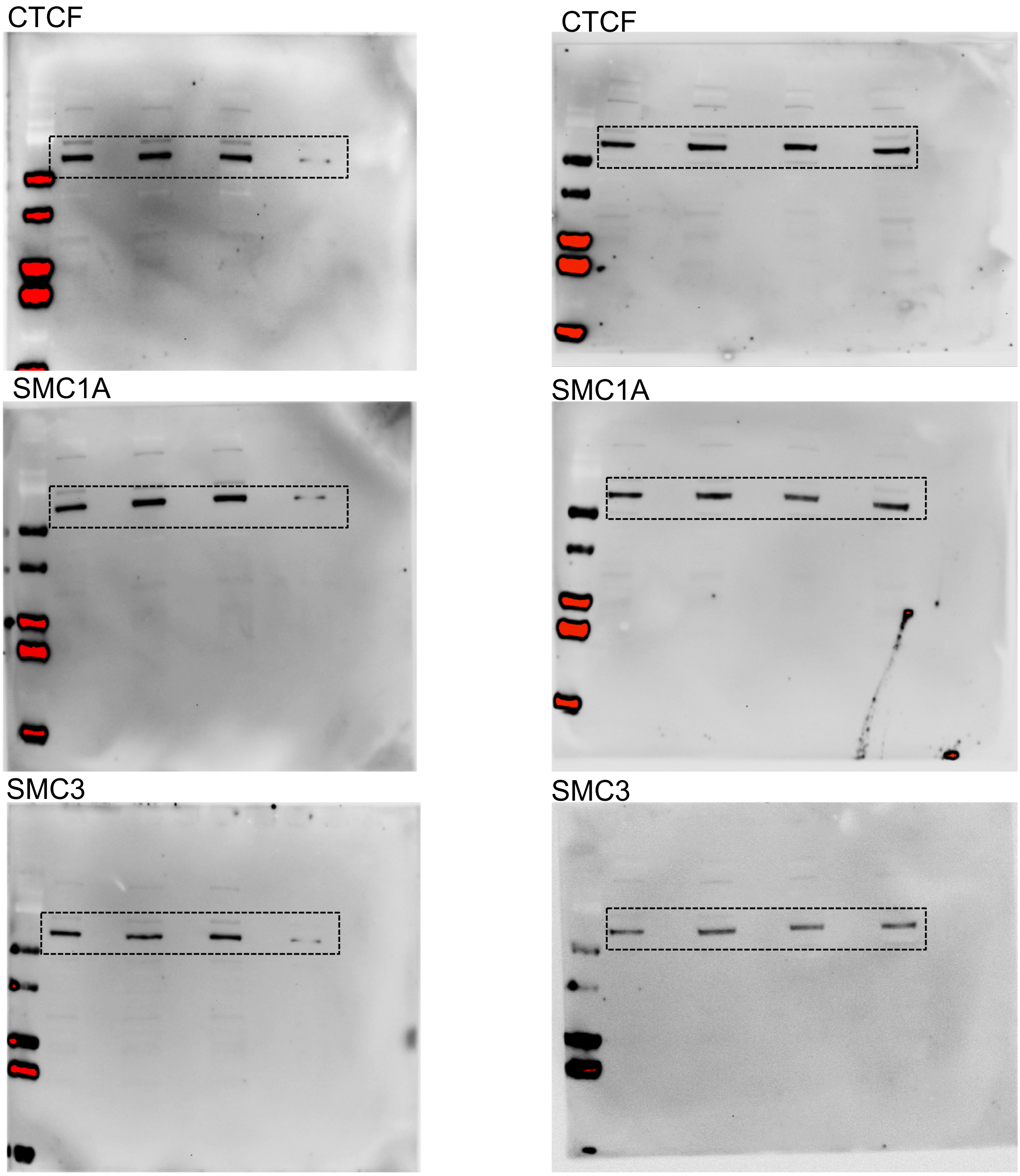

Supplementary Figure 37: Uncropped Western blot images for Figure 8a.

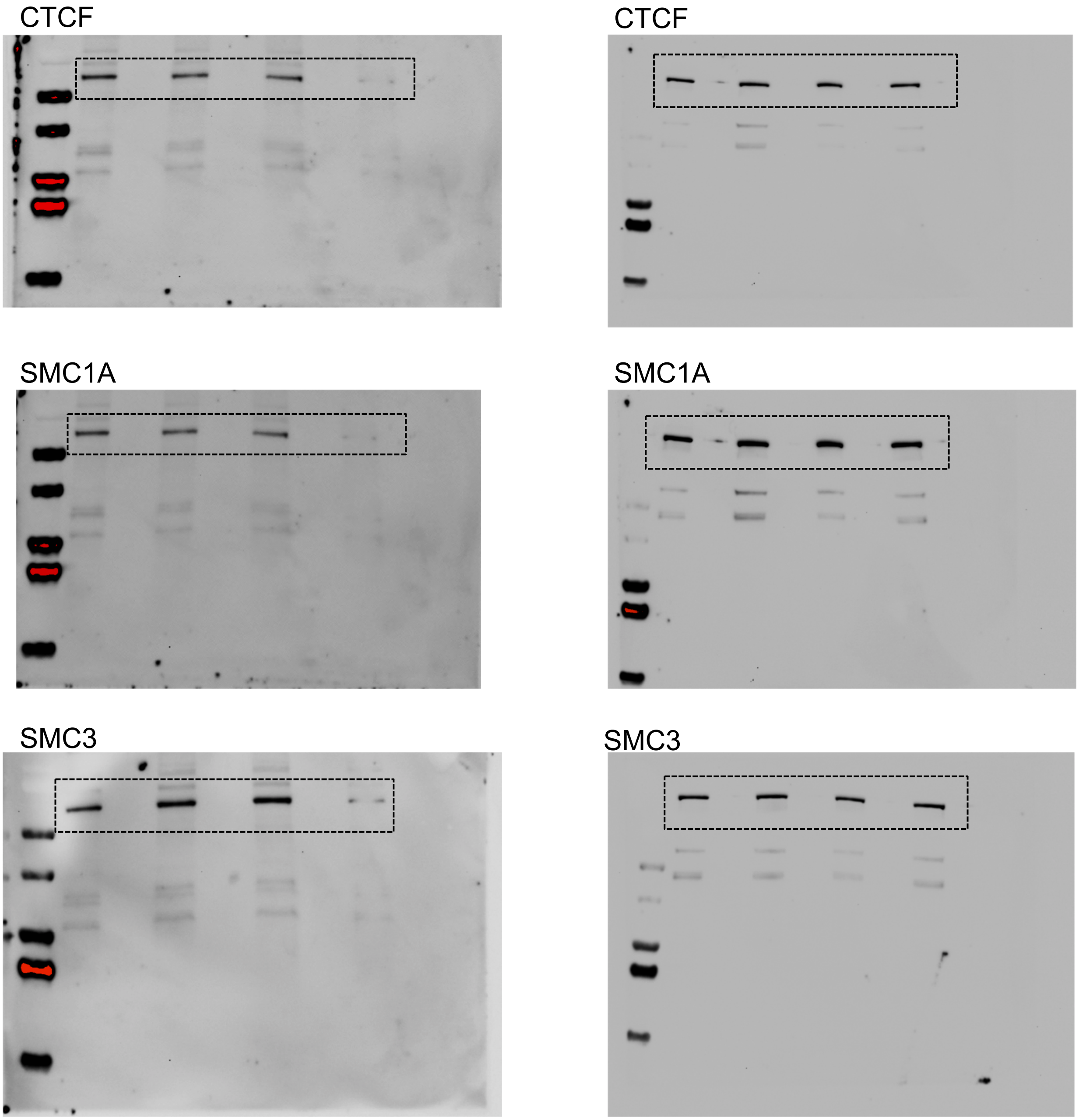

Supplementary Figure 38: Uncropped Western blot images for Figure 8b.

Supplementary Table 1: PCR analyses

Locus	Primers and Probes	Annealing Temp (°C)	Amplicon (bp)	SNP Enzyme [B6/CAST (bp)]	Reference
**Expression Analysis: PCR and/or Restriction Digestion**					
Cdkn1c∆neoR	F 5'GGGAACTTCCTGACTAGGGG 3'	56	284		
	R 5' CCACGTTTGGAGAGGGACAC 3'				
Nup62	F 5'ACCAGTGACCCATTGCAACA3'	58	240		
	R 5'TCAACAACCAACCACGGGAA3'				
Nup98	F 5'TACACCCAGCAGGCATTGTT3'	58	254		
	R 5'TTCACCCACAGGTGGCTTTT3'				
Nup107	F 5'CAGTCTGTGACACGTGGGAA3'	58	341		
	R 5'GAGGTGTGTCATGAAGCGGA3'				
Nup153	F 5' GGGAGTGTCCAGTCTGCTGT3'	58	167		Jacinto et. al. 2015
	R 5' TCCCTCGGGCTTTTTAAACT3'				
Mrpl	F 5' TTGGATATGCCAAGTGACCA 3'	56	212		
	R 5' GCTTCTGCGTTTGAGTTC 3'				
GFP	F 5'GTGACCACCCTGACCTACGG3'	56	150		
	R 5'GATGTTGTGGCGAGGAGGATA3'				

Expression Analysis: Droplet digital PCR primers and probes

Locus	Primers and Probes (Strain)	Annealing Temp (°C)	Amplicon (bp)	Exon-Exon	dbSNP ID
Osbpl5	F 5'AAGTCTCGTCCAGCTCTC3'	60	119	Yes	
	R 5'TCACAGCCGCAAGACCAA3'				
	5'HEX/AGTTCCCCTCC+GTGGTCCA/IBFQ3' (CAST)				rs246186414 / rs263464688
	5'6-FAM/TAGTTCCCCTCC+ATGGCCCA/IBFQ3' (B6)				
Phlda2	F 5'CTTGAGGATGGAGTGGAAA3'	58	160	No	
	R 5'CTCCGACGAGATCCTTTG3'				
	5'HEX/CTTCTTCTTC+CA+GA+CCTGGA/IBFQ3' (CAST)				rs239988740
	5'6-FAM/CTTCTT+CTT+CC+A+TA+CCTGGA/IBFQ3' (B6)				
Slc22a18	F 5'CAGTCCCACAACAGCAAAGAC3'	60	138	Yes	
	R 5'CAGGCTGGCTACCTCATGTC3'				
	5'HEX/ATCGCAGCA+GGGCTTCC/IBFQ3' (CAST)				rs236373969
	5'6-FAM/TCGCAGCA+A+GGCTTCC/IBFQ3' (B6)				
Cdkn1c	F 5'ACACCTTGGGACCAGCGTAC3'	60	363	Yes	
	R 5'GCCAATGCGAACGACTTCTTC3'				
	5'HEX/AGTGT+T+CT+C+A+ATA+CA/IBFQ3' (B6)				rs225291981
	5'6-FAM/TGT+T+CT+C+G+AT+ACA/IBFQ3' (CAST)				
Kcnq1ot1	F 5'CAGCAAGGTTACATGACTCAAA3'	58	109	No	
	R 5'TTGGTGATGCCAAAGCAA3'				rs249486819
	5'HEX/TGCCCCAAG/ZEN/TTAACGAGTGGAA/IBFQ3' (B6)				
	5'6-FAM/TGCCCCAAG/ZEN/TGAACGAGTGG/IBFQ3' (CAST)				
Kcnq1	F 5'TGTTCGCCACATCAGCTATCAG3'	60	118	Yes	
	R 5'TGGCGGTGAATGAAGACTACAG3'				
	5'HEX/CGGATGCTG/ZEN/CATGTAGATCGCC/IBFQ3' (CAST)				rs261791133
	5'6-FAM/CGGATGCT/ZEN/GCATGTCGATCGC/IBFQ3' (B6)				
Tssc4	F 5'GAGTAGAGGCACTTGACACATAG3'	60	98	Yes	
	R 5'CATCCTCGGTGGGTTCATC3'				
	5'HEX/CAT+A+C+GA+C+C+TG/IBFQ3' (B6)				rs221604987
	5'6-FAM/CA+T+A+T+GA+C+CTG/IBFQ3' (CAST)				
Cd81	F 5'GCGTCCTTGCTTCAAAGAGA3'	60	1011	Yes	
	R 5'AAGTGTAGCAGAGTGATACAGAAA3'				
	5'HEX/AAT+GC+A+A+AG+G+GC/IBFQ3' (B6)				rs239612979
	5'6-FAM/AT+GC+A+G+AGGGC/IBFQ3' (CAST)				
Th	F 5'AAATCACGGGCAGACAGTAG3'	60	103	Yes	
	R 5'GAGAGGACAGCATTCCACAG3'				
	5'HEX/AA+G+T+G+AG+A+CAC/IBFQ3' (B6)				rs262280270
	5'6-FAM/AA+G+C+GAG+A+C+AC/IBFQ3' (CAST)				

SNP, single nucleotide polymorphism; F, forward primer; R, reverse primer; Exon-Exon, spans at least one intron; dbSNP ID, SNP database identification number (rs#, reference SNP ID number); HEX, hexachlorofluorescein; 6-FAM, 6-fluorescein amidite; orange nucleotide, SNP between B6 and CAST; +, locked nucleic acid; IBFQ, Iowa Black fluorescein quencher; ZEN, ZEN internal quencher.

Supplementary Table 1: PCR analyses continued

Chromatin Immunoprecipitation

**Locus**	**Primers**	**Annealing Temp (°C)**	**Amplicon (bp)**	**SNP Enzyme [B6/CAST (bp)]**	**Reference**
Osbpl5 Promoter 1 (Os1)	F 5'CCTTTCTCGGCCTTCCAC3'<br>R 5'CCCTCTGGTATTTCCTGCTG3'	60	152	AvaII<br>(101, 51 / 152)	
Osbpl5 Promoter 2 (Os2)	F 5'AGCCAAAGGAGGGGAGGGTGG3'<br>R 5'ATGGACCTGTGATCTTCTGGAA3'	60	296	DdeI<br>(217, 46, 33 / 198, 46, 33, 19)	
Phlda2 Promoter 1 (Ph1)	F 5'GGATGGAGTGGAAAAACAGCTCC3'<br>R 5'GCCCGACTCCGGGGCGTGTCTG3'	60	301	Hpy188III<br>(221, 80 / 301)	
Phlda2 Promoter 2 (Ph2)	F 5'CGATCTCCTTGTAATAGTTGGTGA3'<br>R 5'GACGAGATCCTTTGCGAGGGCGA3'	60	226	Hpy188III<br>(226 / 151, 75)	
Slc22a18 Promoter 1 (Sl1)	F 5'GGACTCTGGACCCCAGGA<br>R 5'TTCCTCTGCCAAGAGGTTGT3'	59	158	BsaJI<br>(102, 56 / 158)	
Slc22a18 Promoter 2 (Sl2)	F 5'GCTTCTCCTTAGGACCCCACTGG3'<br>R 5'GAGGAACAGGGAACCCTAGAACCTG3'	61	188	HpyCH4III<br>(103, 85 / 188)	
Kcnq1ot1 ICR1	F 5' GCCCAGGATTCATAAGCAAG3'<br>R 5' GTTCACAGGACTGGCTCTCC 3'	58		167	
Kcnq1ot1 ICR2	F 5' AAAAGCAATCCACCCTCTCA 3'<br>R 5' GAGTGGCTGGGGTCTAACCT 3'	60		223	
Kcnq1ot1 ICR1 (IC3)	F 5'ATGATAGGCTGGATACAGATAG3'<br>R 5'GTACATTGGCTGCCTCAGGT3'	60	249	BsaJI<br>(171, 78 / 249)	
Kcnq1ot1 ICR2 (IC4)	F 5'TCCCCAACAGAGACCTGAGCC3'<br>R 5'GTTTTCCGATGGTCTTAGGCG3'	60	208	NlaIII<br>(152, 56 / 208)	
Kcnq1ot1 ICR5	F 5'ACAACAAAATGGAGGACTAAGGT 3'<br>R 5' GGTTTTGAGTGTGAGCATAATGG 3'	58		169	
Kcnq1ot1 ICR6	F 5' ACAAGAACAACAAAATGGAGGAC 3'<br>F 5' GTTGGTTTTGAGTGTGAGCATAA 3'	58		178	
Kcnq1ot1 ICR7	R 5' ACAGAAAAGGGAATGTGACCA 3'<br>F 5' AGCATAATGGTCTGAGGTAGGG 3'	58		183	
Kcnq1ot1 ICR8	R 5' GAAAGCACTCCTCCCCATTT 3'<br>F 5' AATGGTCTGAGGTAGGGATCA 3'	60		199	
Kcnq1ot1 ICR9	R 5' CCAGAAAGGTTACAGCGGAA 3'<br>F 5' TCACATTTTGAGACATGGGGT 3'	60		155	
Kcnq1ot1 ICR10	R 5' CAAAGGGCACACGGTATGAG 3'<br>F 5'AAATGGGGAGGAGTGCTTTC 3'	60		175	
Kcnq1ot1 ICR11	R 5' GGCAAGCTCTAGGGAACAGA 3'<br>F 5' CTTTCTGGGCATAAAAGGGAGT 3'	60		197	
Kcnq1ot1 ICR12	R 5' GGGGCAAGCTCTAGGGAAC 3'<br>F 5' TCTGGGCATAAAAGGGAGTAAGT 3'	60		196	
Kcnq1ot1 ICR13	R 5' TAATCCTGCCTGCCTGCTTT 3'<br>F 5' CAATCTTTTCTCATACCGTGTGC 3'	60		154	
Kcnq1ot1 ICR14	R 5' GTTAATCCTGCCTGCCTGC 3'<br>F 5' TTTCTCATACCGTGTGCCCT 3'	60		150	
Kcnq1ot1 ICR15	R 5' CCTTCATCTATGTTCACCAGGG 3'<br>F 5' CTGTTTTGGGATTTGTGCTCC 3'	60		130	
Enhancer element 1 (E1)	F 5'TTCAAACCATCTTTATGCTCCA3'<br>R 5'TGCCCTTAGCTCAAATCCAG3'	58	244	TspDTI<br>(164, 80 / 244)	
Enhancer element 2 (E2)	F 5'AAAGGCTCAGGGAAGCTTTC3'<br>R 5'TTTCCTTTCCCTGTGCTTGC3'	58	198	HindIII<br>(152, 46 / 198)	
Enhancer element 3	R 5' CTGTCCATCTATCCCCGGTC 3'<br>F 5' GGAGCATGAGTTGGAGGAGT 3'	60		193	
Enhancer element 4	R 5' ACAGAGGAAGGCAAGTGTCA 3'<br>F 5' GACCGGGGATAGATGGACAG 3'	58		150	
Enhancer element 5	R 5' GACCCTCCCTGCACATTCTA 3'<br>F 5' AACCAGCCTAGAATGTGCCT 3'	58		161	
Enhancer element 6	R 5' GGGGCTCAGCTGTGTTTAAA 3'<br>F 5' GAGGCCCAAACCAAACAGAG 3'	56		194	

SNP, single nucleotide polymorphism; F, forward primer; R, reverse primer; ICR, imprinting control region.

Supplementary Table 1: PCR analyses continued

Chromatin Immunoprecipitation

**Locus**	**Primers**	**Annealing Temp (°C)**	**Amplicon (bp)**	**SNP Enzyme [B6/CAST (bp)]**	**Reference**
Kcnq1 Promoter 1 (Kc1)	F 5'GATTTTCGCATTCTCCGCCG3'<br>R 5'GCAGATACCACCACCACGAA3'	58	202	SfaI<br>(202 / 144, 88)	
Kcnq1 Promoter 2 (Kc2)	F 5'GCTCCTTCTGTGTCACTCTGG3'<br>R 5'GGACACTGAGCACCCTTGG3'	58	265	AluI<br>(151, 114 / 265)	
Tssc4 Promoter 1 (Ts1)	F 5'ACCTGTTCCGTGCTTGGTT3'<br>R 5'GCCTGTCTGTGCTTCCTGAC3'	57	284	AvaII<br>(284 / 202, 82)	
Th Promoter 1 (Th1)	F 5'GGGTGGGCATAGTGCAAG3'<br>R 5'CCTGGAGGGGACTTGAAGA3'	58	467	BslI<br>(289, 178 / 352, 115)	
Control 1 (Ctrl1)	F 5' TGGCATAGAACCTTCACAACACC3'<br>R 5'CAAATACAGAAGAGAATGCTCACAG 3'	58	287	NlaIII<br>(287 / 222, 65)	
Control 2 (Ctrl2)	F 5'TCTTACAAAGTGCCTTGCTGACC3'<br>R 5'ATTTCTGTGAGTGTGAGTCCAGC3'	58	301	HindIII<br>(189, 112 / 301)	

SNP, single nucleotide polymorphism; F, forward primer; R, reverse primer.

Supplementary Table 2: siRNA transfection details

siRNA Target	Type	Supplier	Catalog	Concentration	Transfection Duration
Nup107-A	SMARTpool ON-TARGETplus	Dharmacon	L-065221-01-0005	10 nmol	48 h
Nup62-A	SMARTpool ON-TARGETplus	Dharmacon	L-064100-01-0005	10 nmol	48 h
Nup98-A	SMARTpool ON-TARGETplus	Dharmacon	L-060137-01-0005	10 nmol	48 h
siNT-A	SMARTpool ON-TARGETplus	Dharmacon	D-001810-01-20	10 nmol	48 h
Nup107-B1	Silencer Select siRNA	Life Technologies	s98083	10 nmol	48 h
Nup107-B2	Silencer Select siRNA	Life Technologies	s98085	10 nmol	48 h
Nup62-B1	Silencer Select siRNA	Life Technologies	s70887	10 nmol	48 h
Nup62-B2	Silencer Select siRNA	Life Technologies	s70885	10 nmol	48 h
Nup98-B1	Silencer Select siRNA	Life Technologies	s114479	10 nmol	48 h
Nup98-B2	Silencer Select siRNA	Life Technologies	s114477	10 nmol	48 h
Nup153-A	Silencer Select siRNA	Life Technologies	s104224	10 nmol	48 h
Nup153-B	Silencer Select siRNA	Life Technologies	s104225	10 nmol	48 h
siNT-B	Silencer Select siRNA	Life Technologies	4390847	10 nmol	48 h

Supplementary Table 3: Antibodies and Probes

Antibody	Catalogue Number	Supplier	Concentration/ Dilution	Duration	Temp (°C)
**Western**					
mAb414	ab24609	Abcam	1:1000	overnight	4
NUP98	ab50610	Abcam	1:1000	overnight	4
NUP107	ab73290	Abcam	1:1000	1 hr	RT
NUP153	sc-101545	Santa Cruz Biotech	1: 1000	1 hr	RT
NUP50	ab137092	Abcam	1:1000	1 hr	RT
NUP93	ab168805	Abcam	1:1000	1 hr	RT
NUP160	ab74147	Abcam	1:1000	1 hr	RT
NUP358	ab2938	Abcam	1:1000	1 hr	RT
ELYS	A300-166A	Bethyl Laboratories	1:1000	1 hr	RT
TPR	sc-271565	Santa Cruz Biotech	1:1000	1 hr	RT
H3	ab1791	Abcam	1:5000	1 hr	RT
INCENP	I5283	Sigma-Aldrich	1:10,000	1 hr	RT
GFP	G46-66M	SignalChem	1:3000	overnight	4
SMC1A	A300-055A	Bethyl Laboratories	1:3000	overnight	4
SMC3	A300-060A	Bethyl Laboratories	1:3000	overnight	4
α-TUBULIN	sc-8035	Santa Cruz Biotech	1:7000	1 hr	RT
Anti-Mouse-HRP Secondary	SC-2314	Santa Cruz Biotech	1:5000	1 hr	RT
Anti-Rat-HRP Secondary	SC-2956	Santa Cruz Biotech	1:4000	1 hr	RT
Anti-Rabbit-HRP Secondary	G33-62G	SignalChem	1:6000	1 hr	RT
**Immunohistochemistry/RNA FISH**					
LaminB1 (S-20)	sc-30264	Santa Cruz Biotech	1:1000	1 hr	37
Anti-Biotin	ab1227	Abcam	1:1000	1 hr	37
Anti-Rabbit Alexa Fluor® 488	A-11034	Life Technologies	1:1000	1 hr	37
Anti-Goat Alexa Fluor® 594	A-11080	Life Technologies	1:1000	1 hr	37
**Chromatin Immunoprecipitation / Immunoprecipitation**					
H3	ab1791	Abcam	2 μg	overnight	4
H3K4me3	39159	Active Motif	3 μg	overnight	4
H3K9me2	ab1220	Abcam	3 μg	overnight	4
H3K27me3	07-449	Millipore	3 μg	overnight	4
RNAPII	sc-889	Millipore	3 μg	3 hr	4
mAb414	ab24609	Abcam	4 μg	overnight	4
NUP98	ab50610	Abcam	4 μg	overnight	4
NUP153	sc-101545	Santa Cruz Biotech	4 μg	overnight	RT
SMC1A	A300-055A	Bethyl Laboratories	1.5 μg	overnight	4
SMC3	A300-060A	Bethyl Laboratories	1.5 μg	overnight	4
KMT2A	39829	Active Motif	3 μg	overnight	
EHMT	ab41969	Abcam	3 μg	overnight	
EZH2	07-689	Millipore	3 μg	overnight	
Anti-Mouse IgG	sc-2029	Santa Cruz Biotech	*	*	*
Anti-Rabbit IgG	2729s	Cell Signalling	*	*	*
Anti-Goat IgG	sc-2028	Santa Cruz Biotech	*	*	*

* same as corresponding antibody used

Supplementary Table 4: List of ChIP primers for supplementary information

Gene	Primers	Annealing Temperature	SNP Enzyme [B6/CAST (bp)]	Amplicon (bp)	Reference
Vim Promoter	F 5' GGTTTCTTGTCCGACTTGCT 3'<br>R 5'CCTCCCCCTTTCTCTCTTTC 3'	58	MluCI<br>(169 / 135, 34)	169	
Vim Exon 3	F 5' GCCTCTGCCAACCTTTTCTT 3'<br>R 5' GGGGACTTTTGGGATAGCAT 3'	58		183	
Vim 3'UTR	F 5' CCTCCCAGTGATGACCAACT 3'<br>R 5' GGAACTGAAGGGGTTTGCAG 3'	60		167	
Orai2 Promoter	F 5'GGTAGAGGCGTGCAAGAAAG 3'<br>R 5'TTCTCTGCCATCCATGAGGT 3'	60	MluCI<br>(185 / 139,46)	185	
Orai2 Exon 2	F 5' GTAAGCCCCAGTGTTCCTCT 3'<br>R 5' GAGATCTGCCTCCTGAGTCC 3'	60		240	
Orai2 3'UTR	F 5' GTGCAGGAGGAGTTGAGAGT 3'<br>R 5' GTGCAGGAGGAGTTGAGAGT 3'	60		189	
Shank2 Promoter	F 5' GAAGTCTACAGGGGACCTTCG 3'<br>R 5' TCCCACATAAGCCTGTCTCTG 3'	60	BfaI<br>(162, 39 / 192)	192	
Shank2 Exon 4	F 5' TGGGAATGTCACTTCCTCCC 3'<br>R 5' ACCTAAGCCACGATTCCACA3'	60		179	
Dhcr7 promoter	F 5' GCCATTTGCTTGTCAGAGGT 3'<br>R 5' CTGGAAGCAGACCAGTTGTG 3'	60	BfI<br>(172 / 127, 45)	172	
Dhcr7 Exon3	F 5' GAAAGGCAAGCTGTTTCCCA 3'<br>R 5' TGGAATGAGCATGGGGTTCT 3'	58		219	
Dhcr7 3'UTR	F 5' AGTCTCTGCCAGTAACCCAC 3'<br>R 5' TGCTGCGTGAGACTGAGTAA 3'	58		243	
H19 ICR	R 5' CAGTTGTGTTTCTGGAGGG3'<br>F 5' TAGGAGTATGCTGCCACC3'	58	DpnII<br>(398 / 300, 98)	137	Verona et. al. 2008
H19 exon5	R 5' GCACTAAGTCGATTGCACTGG3'<br>F 5'AACACTTTATGATGGAACTGC 3'	58	BgII<br>(212 / 124, 88)	212	Verona et. al. 2008
FoxA2 promoter	R 5' TTTCAAGACATCCGGGCTAC 3'<br>F 5' CACGAAATTGTGGACACCTG 3'	58		279	
MyoD promoter	F 5' TCCGCCCTACTACACTCCTA 3'<br>R 5'TGGTGAAGAAAGCAGTCGTG 3'	58		196	

SNP, single nucleotide polymorphism; F, forward primer; R, reverse primer.

Supplementary Table 5: Supplementary PCR analyses

Locus	Primers and Probes	Annealing Temp (°C)	Amplicon (bp)	SNP Enzyme [B6/CAST (bp)]	Reference
**Expression Analysis: PCR and Restriction Digestion**					
Osbpl5	F 5'CAGGGCAGCAGCAGAGGAT3'<br>R 5'GGAGGTTGTTCTTCTCACACTCG3'	57	415	AluI<br>(369, 40, 22, 21 / 319, 90, 40, 21, 22)	Golding et. al. 2011
Phlda2	F 5'ATCCTTTGCGAGGGCGAGC3'<br>GCGTTTCACGGACCCAGA3'	60	389	Hpy188III<br>(389 / 329, 69)	Golding et. al. 2011
Slc22a18	F 5'ATCAACAGGACTTTTGCCCC3'<br>R 5'ATCAACAGGACTTTTGCCCC3'	56	257	SacII<br>(200, 57 / 227, 30)	Golding et. al. 2011
Cdkn1c	F 5'GCCAATGCGAACGACTTC3'<br>R 5'TACACCTTGGGACCAGCGTACTCC3'	58	364	Taq1<br>(306, 58 / 257, 58, 49)	Golding et. al. 2011
Kcnq1ot1	F 5'GATCAGCATGGGTTATTGGA3'<br>R 5'ATTAAGGGACCACAGCAAGG3'	60	213	HpaI<br>(173, 40 / 213)	Korostowski et. al. 2012
Kcnq1	F 5'CATCGGTGCCCGTCTGAACAGG3'<br>R 5'TTGCTGGGTAGGAAGAGCTCAG3'	58	189	NlaIII<br>(92, 76, 21 / 113, 76)	Golding et. al. 2011
Tssc4	F 5'AGAAGCTGCCCATCCTGAGT3'<br>R 5'GACCCACAATTCCCACAGTC3'	58	262	AluI<br>(262 / 223, 39)	Lewis et. al. 2006
Cd81	F 5'GCGTCCTTGCTTCAAAGAGA3'<br>R 5'AGGCAAACAGGATCACAAGG3'	58	510	FauI<br>(510 / 479, 31)	Lewis et. al. 2006
Th	F 5'CTTCCGTGTGTTTCAGTGC3'<br>R 5'ACCGTGGAGAGTTTTTCAA3'	57	195	BsrI<br>(124, 71 / 195)	Golding et. al. 2011
**Bisulfite mutagenesis**					
BIS Outer	F 5'GTGTGATTTTATTTGGAGAG3'<br>R 5'CCACTCACTACCTTAATACTAACCAC3'	52	571		Denomme et al, 2011
BIS Inner	F 5'GGTTAGAAGTAGAGGTGATT3'<br>R5'TACTGAATTTCAAAACCACCCCTACTTGTAT3'	52	228		

SNP, single nucleotide polymorphism; F, forward primer; R, reverse primer; ICR, imprinting control region; BIS, bisulfite mutagenesis.

SUPPLEMENTAL METHODS

RNA stability assay

Kcnq1ot1 half-life studies were performed by addition of 2 mg/mL of actinomycin D (Sigma, A9415) to control and nucleoporin-depleted XEN cells for 4 hours. Cells were collected at 0 hours and every hour time-intervals up to 12 hours. RNA was extracted, and cDNA was synthesized as described in the manuscript. *Kcnq1ot1* ncRNA levels were normalized to time 0 hours for each treatment.

Electromobility Shift Assay

Electromobility shift assay was performed as per the manufacturer's instructions using the LightShift™ Chemiluminescent EMSA Kit (ThermoFisher Scientific, 20148). Nuclear extracts were prepared from XEN cells using NE-PER Nuclear and Cytoplasmic Extraction Kit (ThermoFisher Scientific, 78833) as per the manufacturer's protocol. Nuclear extract equivalent to 100,000 XEN cells (3 μL) were used per EMSA experiment in a 20 μL final volume. PCR amplified DNA fragments were purified using ChIP DNA Clean & Concentrator™ (Zymo Research, D5205) and subjected to biotin end-labeling (ThermoFisher Scientific, 89818). Labeled DNA was incubated with nuclear lysates and target antibody for 20-30 minutes at room temperature followed by loading on a 6% polyacrylamide gel (29:1 Acrylamide solution, BioRad, 1610156) and separation by electrophoresis at 100V for 1 hour. Resolved samples on polyacrylamide gels were transferred onto a nylon membrane (BioRad, 1620159) followed by cross-linking the DNA on the membrane under UV-light. Shift and supershift were then detected using anti-biotin antibody using chemiluminescence. Antibodies that were used for EMSA assay are listed in Supplementary Table 3.

Biotin Immunoprecipitation Assay

Biotin immunoprecipitation assay was performed as described (Hazan-Halevy et al., 2010) with modifications. Briefly, 1 μg biotin-labeled DNA was incubated with 100 μg nuclear lysate for two hours at 37°C temperature with constant rotation. Nuclear lysate with biotin-labeled DNA was then incubated with streptavidin antibody-conjugated beads (ThermoFisher Scientific, 88816) at 4°C for 3 hours with constant rotation. Following washes similar to immunoprecipitation described above, IP samples were mixed with Western blot loading 1X Laemmli buffer and subjected to Western blot analysis. See Supplementary Table 2 for list of antibodies.

Allelic expression analysis

Allelic expression analysis was performed by restriction digestion of PCR product followed by separation on an 8% acrylamide (29:1, Bio-Shop) gel. Densitometry was performed quantify band intensity using QuantityOne 1-D Analysis Software (BioRad). Parental allele-specific expression was determined as a percent of B6 or CAST expression relative to the total expression. Supplementary Table 4 lists SNPs, enzymes and fragment sizes.

Nuclear Transport

For nuclear import experiments, XEN cells were transfected with 3 μg of E47-RFP^{NLS} construct using Lipofectamine2000 (Invitrogen), followed by siRNA transfection. As a positive control, XEN cells were treated with 10 μM of ivermectin (Sigma, I8898) for 48 hours. To access growth rates, ~25 000 cells were seeded on a 6 well plate (Corning, C3506), and then transfected 12 hours later with siRNAs. For GFP mRNA and protein nuclear transport,

recombinant lentiviral particles with shRNA targeting *Luciferase* with a GFP and puromycin resistance gene were generated in HEK293 cells[17]. Following transduction and successful rounds of selection with puromycin, *Luciferase*-GFP-positive XEN cell populations were collected and then used for passive nuclear transport experiments. For nuclear export, biotin-labeled oligo-dT-50 (Life Technologies, custom primer design) was used for polyA-mRNA FISH. Three replicates were performed.

XEN cell growth rate and doubling time

Direct cell counts were performed every 12 hours using a hemacytometer (VWR, 15170-208). Population doubling time (DT) was calculated using the following equation, DT = T ln2/ln (X_E/X_B) where X_B is the cell number at the beginning of the incubation, X_E is the cell number at the end of the incubation time (or collection). Three replicates were performed.

Bisulfite mutagenesis and sequencing

Control and siRNA-treated XEN cells (20% confluent) were seeded on gelatin-coated 6-well dishes. Forty-eight hours after transfection, cells were washed once with 1X PBS (Sigma) followed by a 5-minute incubation with 1X Trypsin-EDTA (Sigma) in PBS. Trypsin was inactivated by addition of RPMI medium. Detached cells were collected and pelleted gently at 200 RCF for 5 minutes, washed and re-suspended in 1X PBS. One percent of cells (~10,000 cells) were embedded into a 2:1 3% LMP agarose (Sigma) and lysis solution [100 mM Tris–HCl, pH 7.5 (Bioshop), 500 mM LiCl (Sigma), 10 mM EDTA, pH 8.0 (Sigma), 1% LiDS (Bioshop), and 5 mM DTT (Sigma), 1 µL of 2mg/ml proteinase K (Sigma), and 1 µL 10% Igepal (Sigma)] as described (Denomme et al. 2011). Briefly, the agarose-sample mixture was then placed on ice for 10 minutes to produce an agarose/lysis bead. Samples were then incubated overnight for 20 hours in SDS lysis buffer [450 µL TE pH 7.5, 50 µL 10% SDS, 1 µL proteinase K] at 50°C, following which lysis buffer was removed and 300 µL of mineral oil was added to the top of each XEN cell/bead mixture. Samples were either processed immediately for bisulfite mutagenesis or frozen at -20°C for a maximum of 5 days. Bisulfite mutagenesis was performed as described for *Kcnq1ot1* amplification in XEN cells (Denomme et al. 2011). Samples were first incubated at 90°C to heat inactivate the proteinase K for 2.5 minutes, then transferred to ice for 10 minutes. To denature DNA, 0.1 M NaOH solution was added at 37°C for 15 minutes. For bisulfite conversion, samples were covered with 300 µL of mineral oil, under which 500 µL of 2.5 M bisulfite solution was added. Samples were incubated for 3.5 hours at 50°C, following which desulfonation was completed in 1 mL of 0.3 M NaOH at 37°C for 15 minutes. Two washes each were performed in TE pH 7.5 and autoclaved water. Negative controls (beads without the embedded XEN cells) were processed with each bisulfite reaction. For first round PCR amplification, the XEN cell agarose bead with bisulfite converted DNA (10 µL) was added to 15µL of Hot Start Ready-To-Go PCR bead (GE Healthcare) containing 0.2 µM *Kcnq1ot1* external primers, 9.6 ng/mL transfer RNA with a 25µL mineral oil overlay. For second round PCR, first round PCR product (5 µL) was added to PCR beads containing 0.2 µM *Kcnq1ot1* internal primers. See Supplementary Table 4 for primers. PCR products were ligated into pGEM-Easy vector (Promega) as per the manufacturer's instructions and sequenced at the BioBasic Sequencing Facility (Markham, Canada). Sequences with less than 90% conversion were excluded. Percent methylation was calculated as the number of methylated CpGs over the total number of CpGs.

SUPPLEMENTAL REFERENCES

1. Baldwin AS. 1996. The NF-kappa B and I kappa B proteins: new discoveries and insights. *Annu Rev Immunol* **14**: 649–683.

2. Denomme MM, Zhang L, Mann MRW. 2011. Embryonic imprinting perturbations do not originate from superovulation-induced defects in DNA methylation acquisition. *Fertil Steril* **96**: 734-738.

3. Golding, M. C. *et al.* Depletion of Kcnq1ot1 non-coding RNA does not affect imprinting maintenance in stem cells. *Development* **3678,** 3667–3678 (2011).

4. Hazan-Halevy I, Harris D, Liu Z, Liu J, Li P, Chen X, Shanker S, Ferrajoli A, Keating MJ, Estrov Z. 2010. STAT3 is constitutively phosphorylated on serine 727 residues, binds DNA, and activates transcription in CLL cells. *Blood* **115**, 2852–2863.

5. Jacinto, F. V, Benner, C. & Hetzer, M. W. The nucleoporin Nup153 regulates embryonic stem cell pluripotency through gene silencing. *Genes Dev.* **29,** 1–15 (2015).

6. Korostowski, L., Raval, A., Breuer, G. & Engel, N. Enhancer-driven chromatin interactions during development promote escape from silencing by a long non-coding RNA. *Epigenetics Chromatin* **4,** 21 (2011).

7. Lewis, A. *et al.* Epigenetic dynamics of the Kcnq1 imprinted domain in the early embryo. *Development* **133,** 4203–4210 (2006).

8. Verona, R. I., Thorvaldsen, J. L., Reese, K. J. & Bartolomei, M. S. The transcriptional status but not the imprinting control region determines allele-specific histone modifications at the imprinted H19 locus. *Mol. Cell. Biol.* **28,** 71–82 (2008).
